# Emodin inhibits colon cancer tumor growth by suppressing tumor cell glycolysis through inhibition of NAT10-mediated PGK1 ac4C modification

**DOI:** 10.3389/fonc.2025.1575391

**Published:** 2025-12-05

**Authors:** Zhuoxin Wei, Zhuo Lv, Weimin Zhang

**Affiliations:** 1 Southern Medical University Hospital of Integrated Traditional Chinese and Western Medicine, Southern Medical University, Guangzhou, Guangdong, China; 2 Oncology Department, Guangzhou Hospital of Integrated Traditional and Western Medicine, Guangzhou, Guangdong, China

**Keywords:** colon cancer, emodin, glycolysis, NAT10, ac4C, PGK1

## Abstract

**Introduction:**

Inhibition of glycolysis represents a potent therapeutic approach for colon cancer. Emodin, a natural plant-derived compound with anticancer properties, has shown efficacy in cancer treatment. NAT10 promotes cancer progression by catalyzing ac4C production on mRNAs, but it remains unclear whether emodin regulates glycolysis in colon cancer cells by targeting NAT10. This study aimed to investigate the role of emodin in glycolysis regulation in colon cancer and its underlying mechanisms.

**Methods:**

Glycolysis was assessed by measuring cell proliferation, glucose uptake, lactate production, and extracellular acidification rate. The ac4C levels and NAT10 expression were measured by dot blot and quantitative real-time PCR. The underlying mechanism was investigated by methylated RNA immunoprecipitation (MeRIP), RIP and dual luciferase reports. The effect of emodin on tumor growth in vivo was evaluated by hematoxylin and eosin staining and immunohistochemistry staining.

**Results:**

Results showed that emodin inhibited glycolysis in colon cancer cells in a dose-dependent manner, and suppressed the ac4C levels and NAT10 expression of cells. Moreover, NAT10 overexpression restored glycolysis in colon cancer cells inhibited by emodin. Mechanistically, NAT10 promotes glycolysis of colon cancer cells by stabilizing PGK1 expression through enhancing ac4C modification on PGK1. In vivo experiments suggested that emodin inhibited colon cancer tumor growth, as well as NAT10 and PGK1 expression.

**Discussion:**

In conclusion, we demonstrated that emodin inhibited tumor growth of colon cancer by suppressing glycolysis in tumor cells through inhibiting NAT10-mediated ac4C modification of PGK1, indicating that emodin is an effective medicine for treatment of colon cancer.

## 1 Introduction

Colon cancer is a common malignant tumor of the digestive tract. The early stages of colon cancer typically present with no symptoms, leading to a diagnosis at an advanced stage, thereby contributing to its status as one of the most lethal cancers worldwide (1). Although numerous studies have established the relationship between colon cancer and poor diet, microorganisms, and their metabolites, the precise mechanisms underlying colon cancer remain incompletely elucidated (2). Due to the increased metabolic demand for energy during tumor proliferation, glycolysis-a high-rate but low-yield ATP-producing pathway-is more active in cancer cells (3). Some studies have observed enhanced glycolysis in colon cancer, a metabolic alteration that facilitates the proliferation of cancer cells (4–6). Notably, there is evidence that exogenous inhibition of glycolysis suppresses the tumor growth of colon cancer (7). These findings suggest that suppression of glycolysis may be an effective therapeutic strategy to prevent the development of colon cancer. Therefore, the identification of drugs with potent inhibitory effects on glycolysis is crucial for the treatment of colon cancer.

Emodin is a natural anthraquinone derivative derived from plants and exhibits potent anti-inflammatory, anticancer, and antifibrotic properties (8, 9). Emodin its exerts anti-cancer effects through a variety of mechanisms, including inducing apoptosis, regulating autophagy and enhancing ROS accumulation (10–12). Emodin has been confirmed to effectively prevent the development of breast and renal cancer by inhibiting glycolysis (13, 14). Additionally, several studies have revealed the potential and efficacy of emodin in treating colon cancer (15, 16). However, whether emodin inhibits the development of colon cancer by regulating glycolysis has not been reported.

N4-acetylcytidine (ac4C) is a conserved nucleoside present on tRNA, rRNA and human mRNA, which is catalyzed by N-Acetyltransferase-like protein 10 (NAT10). NAT10 is the only identified writer of ac4C modification, which mediates the progression of multiple diseases by promoting mRNA stability and translation efficiency (17). The role of NAT10 in cancer progression is significant, as it contributes to cancer development through acetylating mRNAs (18, 19). Compelling evidence suggesting that NAT10 plays a crucial role in the pathogenesis of various cancers by modulating glycolysis and has been shown to facilitate colon cancer progression through the inhibition of ferroptosis (20, 21). However, whether NAT10 mediates colon cancer development through glycolysis remains unclear.

In the present study, we investigated the effect of emodin on inhibiting glycolysis in colon cancer and its underlying mechanism. This study may provide a new therapeutic target for the treatment of colon cancer by emodin.

## 2 Methods

### 2.1 Cell culture and treatment

**Figure 1:**
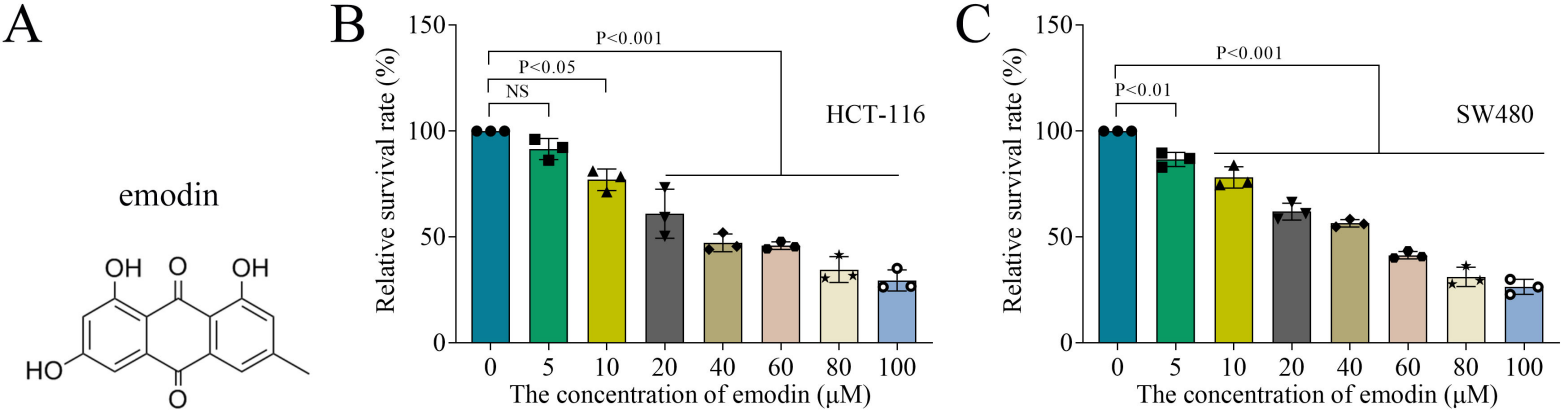
Emodin inhibited cell viability of colon cancer cells. **(A)** The molecular structure of emodin. **(B, C)** Cell viability of HCT-116 and SW480 cells was measured by MTT assay. N = 3 per group. Data analysis was performed using ANOVA.

Colon cancer cell lines HCT-116 and SW480 were provided by Procell (Wuhan, China). HCT-116 cells and SW480 cells were cultured in McCoy’s 5A medium (Gibco, Grand Island, NY, USA) and Leibovitz’s L-15 medium (Gibco) containing 10% fetal bovine serum (FBS; Gibco) and 1% penicillin/streptomycin, respectively at 37°C and 5% CO_2_. To assessed the effect of emodin on colon cancer cell, HCT-116 cells and SW480 cells were treated with different concentrations of emodin for 24 h. The molecular structure of emodin is presented in **Figure 1A**.

### 2.2 Cell transfection

NAT10 overexpressing plasmids (pcDNA3.1-NAT10), PGK1 overexpressing plasmids (pcDNA3.1-PGK1), empty vector (pcDNA3.1), short hairpin RNA targeting NAT10 (shNAT10) and shRNA negative control (shNC) were provided by GenePharma (Shanghai, China). These plasmids were transfected into HCT-116 or SW480 cells using Lipofectamine 2000 (Invitrogen, Carlsbad, CA, USA) following the manufacturer’s protocol for 48 h of transfection.

### 2.3 Cell viability assay

Cell viability was detected by MTT assay. HCT-116 and SW480 cells were seeded into a 96-well plate (2 × 10^3^ cells/well). Emodin at 0, 5, 10, 20, 40, 60, 80, and 100 μm was added to the wells for 24 or 48 h incubation. Next, MTT reagent (Beyotime, Shanghai, China) was added to each well and incubated for 4 h. The supernatant was discarded, and dimethyl sulfoxide (DMSO) was employed for formazan solubilization. The absorbance was measured using a microplate reader.

### 2.4 Cell proliferation assay

Cell proliferation was detected using a BeyoClick™ EdU cell proliferation kit (Beyotime). Cells were seeded in a six-well plate and mixed with EdU solution for 2 h incubation. Next, cells were fixed with 4% paraformaldehyde for 15 min and incubated with 1 mL PBS containing 0.3% Triton X-100 for 15 min. PBS was discarded and cells were incubated with 0.5 ml prepared Click solution. Nucleus was stained using Hoechst 33342. Cells were observed using a fluorescence microscope.

### 2.5 Glucose uptake assay

Glucose uptake was performed using a glucose uptake assay kit (Abcam, Cambridge, MA, USA). Cells were seeded in a 96-well plate and starve in 100 µL serum free medium overnight to increase glucose uptake, and then transferred to 100 µL KRPH buffer containing 2% BSA for 40 min. Following experiments were conducted according to the manufacturer’s protocol and the absorbance was measured at 412 nm using a microplate reader.

### 2.6 Lactate production assay

Lactate production was detected using a lactate content detection kit (Solarbio, Beijing, China). Experiments were conducted according to the manufacturer’s protocol and the absorbance was measured at 570 nm using a microplate reader.

### 2.7 Real-time cell metabolism assay

Extracellular acidification rate (ECAR) of HCT-116 and SW480 cells was measured using an ECAR fluorometric assay kit (Elabscience, Wuhan, China). Cells were centrifuged at 500×g and 4°C for 5 min. The supernatant was discarded and cells were resuspended in reagent I and the cell density was adjusted to 5×10^3^/μl. Cells were incubated for 30 min protected from light. EACR was measured according to the manufacturer’s protocol. The excitation wavelength and the emission wavelength of the fluorescein were set to 490 nm and 535 nm, respectively.

### 2.8 Western blot

Total proteins were extracted from cells and transplanted tumor using RIPA lysis buffer (Beyotime). The protein concentration was determined using a BCA kit (Beyotime). The protein samples were separated by 10% SDS-PAGE and then electrophoretically transferred onto a PVDF membrane. The membrane was blocked with 5% non-fat milk for 1 h at room temperature and subsequently incubated with anti-NAT10 (1: 1000, ab194297, Abcam, Cambridge, MA, USA), anti-HK2 (1: 1000, ab209847, Abcam), anti-PFK1 (1: 1000, PC3982, Abmart, Shanghai, China) and anti-β-actin (1: 5000, ab8227, Abcam) overnight at 4 °C. Subsequently, the membrane was incubated with an HRP-conjugated secondary antibody (1: 10000, ab6721, Abcam) for 1 h at room temperature. Protein bands were visualized using an ECL substrate (Beyotime) and captured with a chemiluminescence imaging system.

### 2.9 Dot blot assay

Total RNA from HCT-116 and SW480 cells were isolated using Trizol reagent (Gibco) and mixed with incubation buffer and denatured at 65°C  for 5 min. The samples were subsequently deposited on an Amersham Hybond-N+ membrane (GE Healthcare, USA) in SSC buffer and subjected to 5 min of UV crosslinking. Then, samples were washed with PBST and stained with 0.02% Methylene blue (Sangon Biotech, China). Input RNA content was displayed by scanning the blue dot. The membrane was incubated overnight at 4°C with anti-ac4C, followed by visualization of dot blots using the imaging system after incubation with a secondary antibody.

### 2.10 Quantitative real-time PCR

Isolated RNA of HCT-116 and SW480 cells was quantified using Nanodrop 2000 (Thermo Scientific, Waltham, MA, USA) and reverse-transcribed into cDNA using HiScript II 1st strand cDNA synthesis kit (Vazyme, Nanjing, China). qPCR was performed using SYBR green (Vazyme) on ABI 7500 (Thermo Scientific). The expression was quantified using the 2-ΔΔCt method with GAPDH serving as the internal reference. The primers used for qPCR were as follows: NAT10, 5’-ATAGCAGCCACAAACATTCGC-3’ (forward) and 5’-ACACACATGCCGAAGGTATTG-3’ (reverse); PGK1, 5’-TGGACGTTAAAGGGAAGCGG-3’ (forward) and 5’-GCTCATAAGGACTACCGACTTGG-3’ (reverse); GAPDH, 5’-GGAGCGAGATCCCTCCAAAAT-3’ (forward) and 5’-GGCTGTTGTCATACTTCTCATGG-3’ (reverse).

### 2.11 Molecular docking

The 3D structure of NAT10 was retrieved from the UniProt database and subsequently optimized geometrically using the Maestro 11.9 platform. The molecular structure of emodin was obtained from the PubChem database, formatted, and energy-minimized using Chem3D. Molecular docking was performed using the Glide module in Schrödinger Maestro software. Protein preparation was conducted using the Protein Preparation Wizard module. Standard precision settings were employed for molecular docking and screening.

### 2.12 Surface plasmon resonance analysis

SRP analysis was performed to detect the binding between emodin and NAT10. Producers were conducted using a Biacore S200 instrument (Cytiva, Marlborough, MA, USA), with data were analyzed using Biacore software (Cytiva). Single-cycle kinetic runs were carried out to evaluate binding kinetics. Curve baselines were adjusted to zero and aligned with the injection start time, while the reference sensorgram was subtracted from the experimental sensorgrams to correct for non-specific binding. Binding kinetics were assessed using a 1:1 binding model.

### 2.13 Methylated RNA immunoprecipitation

The ac4C levels of PGK1 of HCT-116 and SW480 cells were detected by GenSeq^®^ ac4C RIP kit (Cloudseq, Shanghai, China). Total RNA was mixed with fragmentation buffer and fragmented on a PCR instrument at 70°C  for 6 min. PGM magnetic beads were mixed with anti-ac4C for 1 h of incubation at room temperature and washed with IP buffer. Fragmented RNA was added to prepared PGM magnetic beads and incubated for 3 h at 4°C. PGM magnetic beads were washed with IP buffer after centrifugation, and RNA was bound to the magnetic beads. RNA was purified and the ac4C levels of cells was measured by qPCR.

### 2.14 Bioinformatic analysis

The ac4C modification site of PGK1 was predicted utilizing the PACES database (http://rnanut.net/paces/action.php).

### 2.15 RIP

RIP assay was performed to identify the interaction between NAT10 and PGK1 using a RIP assay kit (Beyotime). Protein A/G Agarose was incubated with anti-PGK1 or anti-IgG for 30 min. Protein A/G Agarose pre-coated with antibodies were collected by centrifugation and incubated with cell lystate of HCT-116 cells for 4 h at 4°C. Then, samples were eluted and purified and the expression of PGK1 was measured by qPCR.

### 2.16 Dual luciferase reports

Wild type (WT)-PGK1 and mutation (MUT)-PGK1 plasmids containing ac4C modification sites were cloned into pGL3 vector. HCT-116 cells with 80% cell fusion rate were co-transfected with the luciferase plasmids and either shNC or shNAT10 using Lipofectamine 2000 following the manufacturer’s protocol for 48 h of transfection. The luciferase activity was quantified using a dual-luciferase reporter assay system (Promega, San Luis Obispo, CA, USA).

### 2.17 RNA stability assay

The stability of PGK1 mRNA was evaluated by quantifying the expression levels of PGK1 in HCT-116 cells treated with 5 μg/mL actinomycin D (Merck, Darmstadt, Germany) for 0, 4, 8, and 12 h.

### 2.18 Animal study

*In vivo* anti-tumor study was performed by transplanted tumor model of colon cancer in BALB/c nude mice. Mice were randomly divided into the control group and the emodin group (six mice/group). The drug dosage administered to the nude mice was based on the method of Gu et al. (22) Mice in the emodin group received oral administration (p.o.) of emodin (40 mg/kg) for 7 days, and HCT-116 cells (2×10^6^ cells) were subcutaneously injected into the inguinal region of mice. The tumor volume was measured when volume reached approximately 50 mm^3^. Tumor volumes were measured weekly from 0 to 4 weeks using a vernier caliper and calculated as V = (length) × (width) × (height) × 0.52. Four weeks later, mice were euthanized using 160 mg/kg sodium pentobarbital and tumors were removed for subsequent experiments.

### 2.19 Hematoxylin and eosin staining

Tumors were fixed in 4% paraformaldehyde, dehydrated and made into 5μm of paraffin sections. HE staining was performed using a HE staining kit (Beyotime) following the manufacturer’s protocol. The sections were observed under a microscope.

### 2.20 Immunohistochemistry staining

Paraffin sections received dewax, antigen retrieval and endogenous peroxidase blocking according to the manufacturer’s protocol of M&R HRP/DAB detection IHC kit (Vazyme). The sections were incubated with anti-NAT10 (ab194297, 1: 500, Abcam) or anti-PGK1 (ab154613, 1: 500, Abcam) overnight at 4°C, and then incubated with HRP polymer for 20 min at room temperature. The sections were stained with DAB solution for 5 min at room temperature and then counterstained with hematoxylin. The sections were sealed with neutral gum observed under a microscope.

### 2.21 Statistical analysis

All data were processed and analyzed using SPSS 22.0 software. Results were presented as mean ± SD for at least three replicates. Comparison between two or more groups was performed using student’s t-test or one-way analysis of variance (ANOVA). P<0.05 was recognized as statistically significant.

## 3 Results

### 3.1 Emodin inhibits cell viability of colon cancer cells

To evaluate the effect of emodin on colon cancer cells, HCT-116 and SW480 cells were treated with 0, 5, 10, 20, 40, 60, 80 or 100 μM emodin, and cell viability was measured after 48 h of treatment. As the concentration of emodin increased, the cell viability of HCT-116 and SW480 cells decreased (**Figures 1B, C**). Due to the stronger inhibition of cell viability and cytotoxicity of 40, 60, 80 and 100 μM emodin on colon cancer cells, we chose 5, 10 and 20 μM emodin for the following experiments.

### 3.2 Emodin inhibits cell proliferation and glycolysis in colon cancer cells

**Figure 2:**
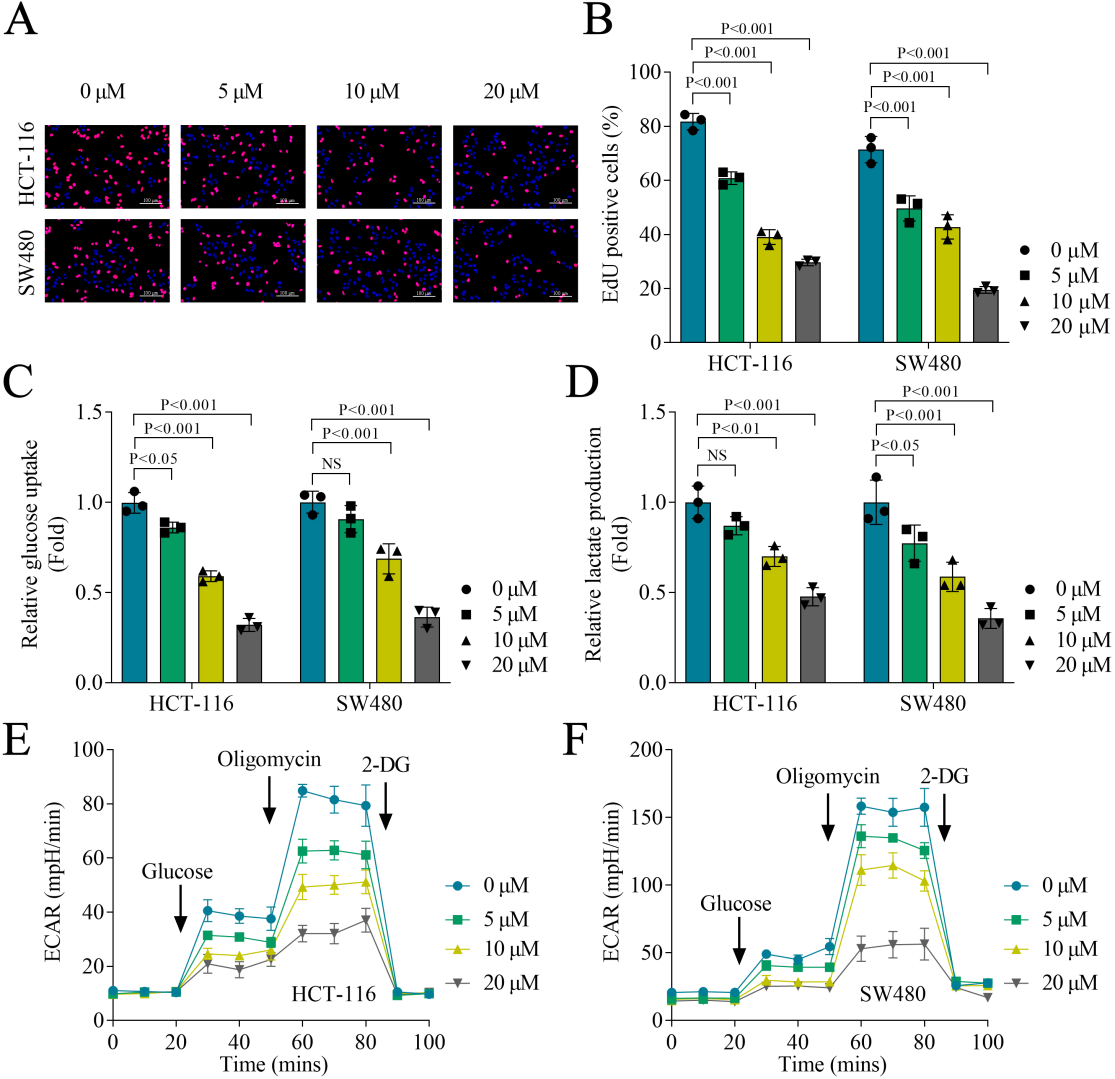
Emodin inhibited cell proliferation and glycolysis in colon cancer cells. **(A, B)** Cell proliferation was evaluated by EdU staining. **(C)** Glucose uptake of HCT-116 and SW480 cells was measured using a glucose uptake assay kit. **(D)** Lactate production was evaluated using a lactate content detection kit. **(E, F)** Real-time cell metabolism was assessed by measuring ECAR. N = 3 per group. Data analysis was performed using ANOVA.

Next, we assessed the effect of different concentrations of emodin on glycolysis in colon cancer cells. Compared with the 0 μM emodin treatment, treatments with 5, 10 and 20 μM emodin significantly inhibited cell proliferation of HCT-116 and SW480 cells, with 20 μM emodin had the strongest suppression effect (**Figures 2A, B**). Similarly, 5, 10 and 20 μM emodin all suppressed glucose uptake, lactate production and ECAR of HCT-116 and SW480 cells, with the most pronounced effects observed at 10 and 20 μM. Moreover, 20 μM emodin exhibited more significant inhibition than 10 μM emodin (**Figures 2C–F**). These results indicated that emodin inhibited glycolysis of colon cancer cells, with the strongest inhibition effect at 20 μM. Therefore, all subsequent cellular experiments were performed using 20 μM emodin. Furthermore, when HCT-116 or SW480 cells were treated with N-Acetylcysteine (NAC), a reactive oxygen species (ROS) scavenger, or dexamethasone (DXM), an anti-inflammatory agent, in the presence of emodin, no restoration of the emodin-suppressed glucose uptake, lactate production, or ECAR was observed. These results indicated that the inhibitory effect of emodin on glycolysis was independent of ROS signaling and inflammatory pathways (**Supplementary Figures S1A–C**).

### 3.3 Emodin suppresses ac4C modification by inhibiting NAT10 mRNA expression in colon cancer cells

**Figure 3:**
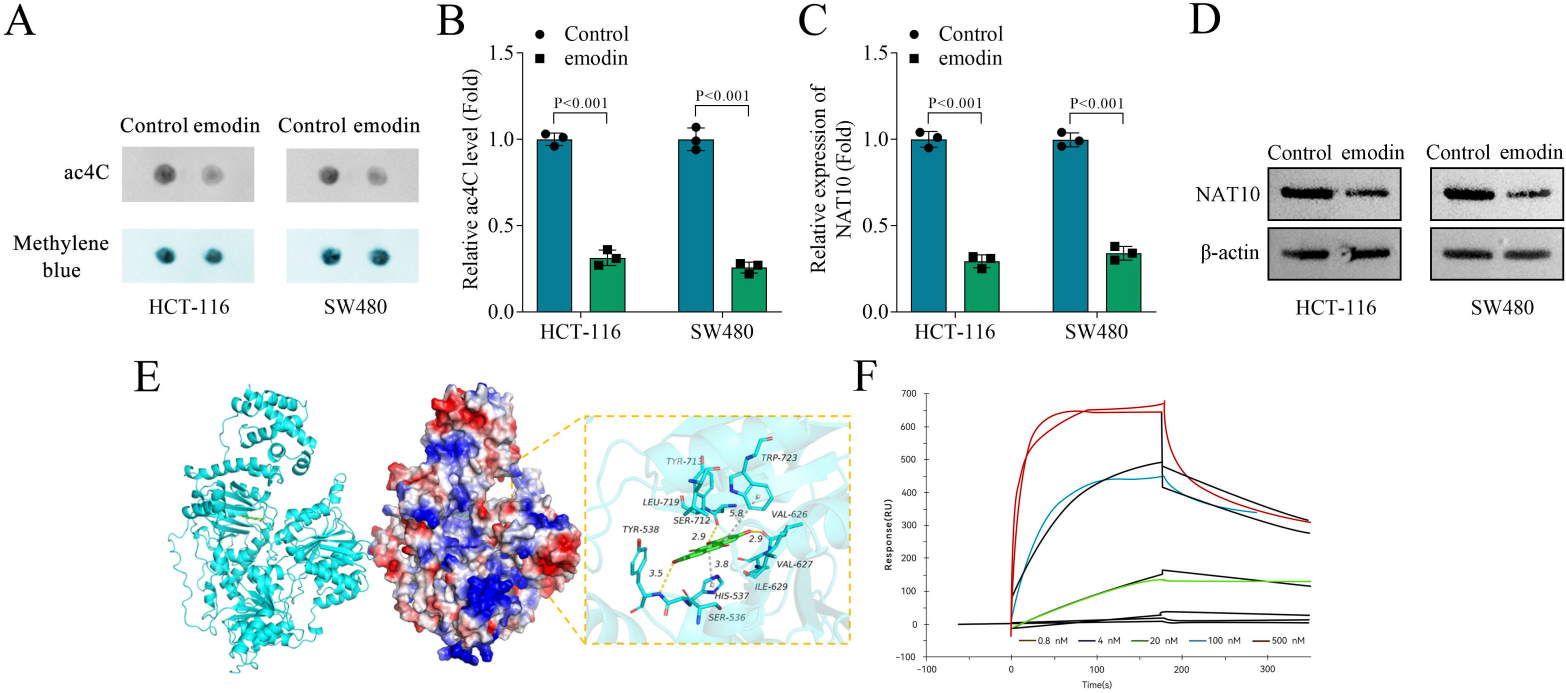
Emodin suppressed ac4C modification by inhibiting NAT10 expression in colon cancer cells. **(A, B)** The ac4C levels of HCT-116 and SW480 cells were detected by dot blot assay. **(C)** The expression of NAT10 in HCT-116 and SW480 cells was measured by qPCR. **(D)** The protein levels of NAT10 in HCT-116 and SW480 cells were detected by western blot. The interaction between NAT10 and eomdin was determined by **(E)** molecular docking and **(F)** SRP assay. N = 3 per group. Data analysis was performed using student’s t-test.

Subsequently, we detected ac4C modification and NAT10 levels in colon cancer cells. Compared with the control group, we found that emodin decreased the ac4C levels (**Figures 3A, B**) and significantly reduced NAT10 mRNA expression and protein levels in both HCT-116 and SW480 cells (**Figures 3C, D**). Molecular docking suggested an interaction between emodin and NAT10 (**Figure 3E**). Moreover, SRP assay suggested that emodin bound to NAT10 (KD = 0.82 μM; **Figure 3F**). Collectively, these results demonstrated that emodin suppressed ac4C modification by inhibiting NAT10 mRNA expression in colon cancer cells.

### 3.4 NAT10 overexpression restores glycolysis inhibited by emodin in colon cancer cells

**Figure 4:**
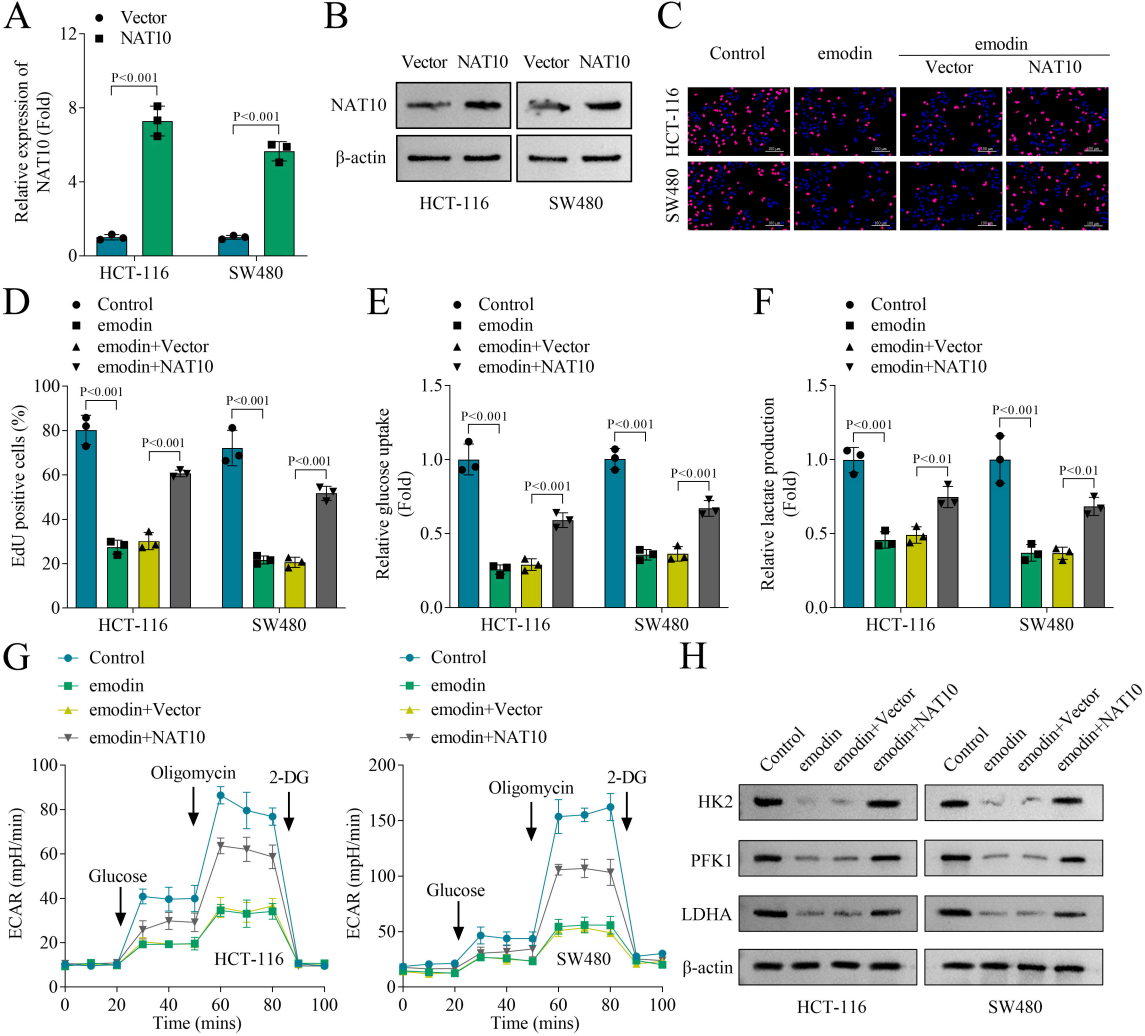
NAT10 overexpression restored glycolysis inhibited by emodin in colon cancer cells. **(A)** The expression of NAT10 was measured by qPCR. **(B)** The protein levels of NAT10 were detected by western blot. **(C, D)** Cell proliferation of HCT-116 and SW480 cells was evaluated by EdU staining. **(E)** Glucose uptake was evaluated using a glucose uptake assay kit. **(F)** Lactate production was evaluated using a lactate content detection kit. **(G)** Real-time cell metabolism was assessed by measuring ECAR. **(H)** The protein levels of HK2, PFK1 and LDHA were detected by western blot. N = 3 per group. Data analysis was performed using student’s t-test **(A)** or ANOVA **(D–G)**.

Next, rescue experiments were performed to determine the role of NAT10 in glycolysis in colon cancer cells. NAT10 mRNA expression and protein levels were significantly upregulated in HCT-116 and SW480 cells transfected with NAT10 overexpressing plasmids (**Figures 4A, B**). NAT10 overexpression enhanced cell proliferation suppressed by emodin of HCT-116 and SW480 cells (**Figures 4C, D**). Moreover, the decreased glucose uptake, lactate production and ECAR of HCT-116 and SW480 cells caused by emodin treatment were restored by NAT10 overexpression (**Figures 4E–G**). Additionally, emodin downregulated the protein levels of key glycolytic upstream enzymes, HK2 and PFK1, in HCT-116 and SW480 cells; moreover, LDHA protein levels in the cells were also reduced by emodin treatment. However, this effect was reversed upon NAT10 overexpression (**Figure 4H**). In conclusion, we demonstrated that NAT10 overexpression restored cell proliferation and glycolysis inhibited by emodin in colon cancer cells.

### 3.5 NAT10 knockdown suppresses ac4C modification on PGK1 by inhibiting its mRNA stability

**Figure 5:**
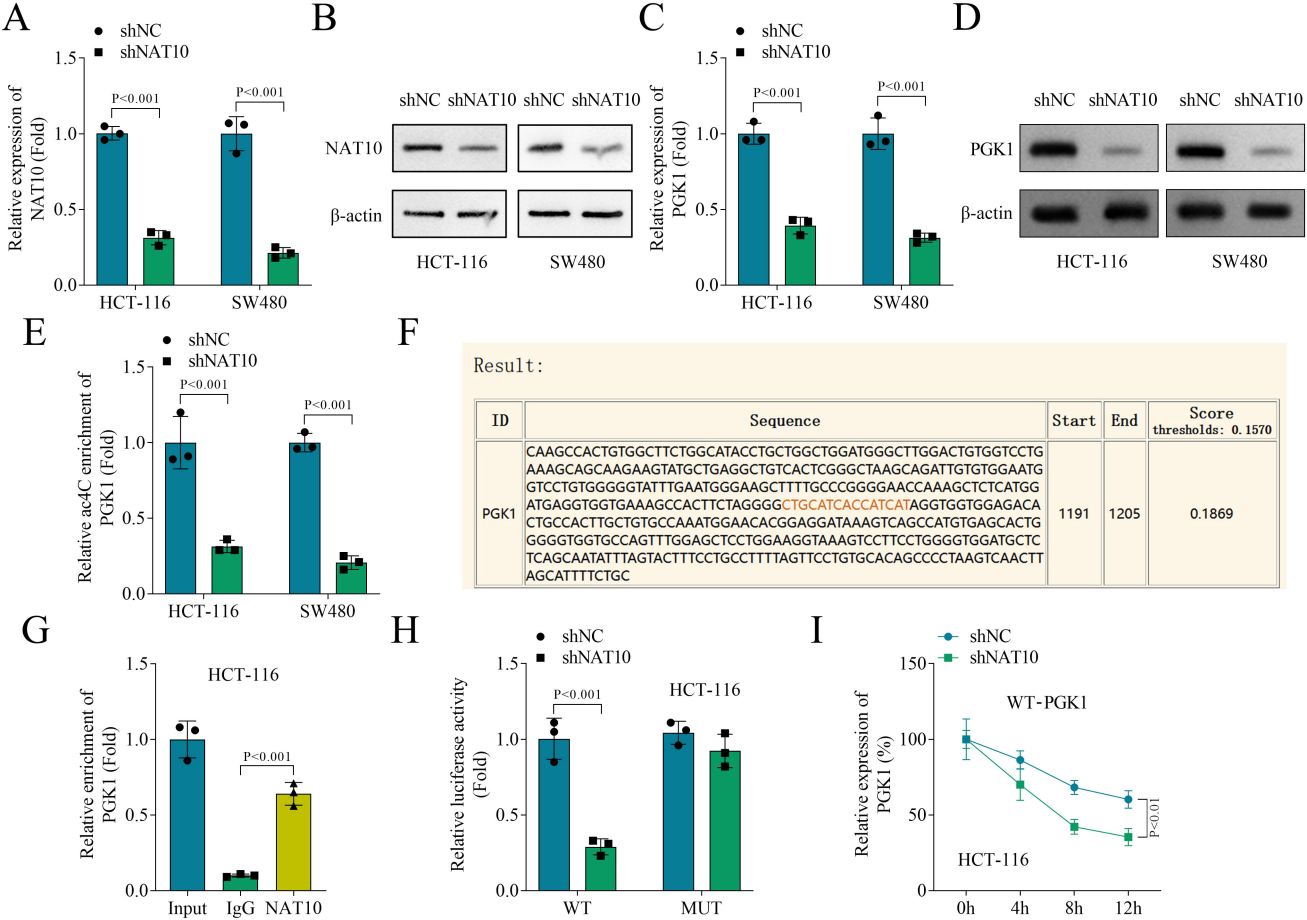
NAT10 knockdown suppressed PGK1 mRNA stability by inhibiting its ac4C modification. **(A)** The expression of NAT10 was measured by qPCR. **(B)** The protein levels of NAT10 were detected by western blot. **(C)** The expression of PGK1 was measured by qPCR. **(D)** The protein levels of PGK1 were detected by western blot. **(E)** The ac4C levels of HCT-116 and SW480 cells were detected by MeRIP. **(F)** The ac4C modification site of PGK1 was predicted using the PACES database. **(G)** The interaction between NAT10 and PGK1 was identified by RIP. **(H)** The luciferase activity of WT-PGK1 and MUT-PGK1 of HCT-116 cells. **(I)** The stability of PGK1 mRNA was measured by qPCR after HCT-116 cells treated with 5 μg/mL actinomycin D for 0, 4, 8, and 12 h. N = 3 per group. Data analysis was performed using student’s t-test **(A, C, D, G)** or ANOVA **(F, H)**.

PGK1 plays an essential role in regulating glycolysis and is involved in the progression of cancers(22). However, whether PGK1 is mediated by NAT10 in colon cancer remain unclear. NAT10 mRNA expression was decreased in HCT-116 and SW480 cells after transfection of shNAT10 (**Figure 5A**). Moreover, NAT10 knockdown decreased the protein levels of NAT10 in HCT-116 and SW480 cells (**Figure 5B**). Next, we found that NAT10 knockdown reduced PGK1 mRNA expression and protein levels in HCT-116 and SW480 cells (**Figures 5C, D**). Moreover, NAT10 knockdown decreased the ac4C modification of PGK1 in HCT-116 and SW480 cells (**Figure 5E**). The predicted ac4C modification site of PGK1 is shown in **Figure 5F**. The interaction between NAT10 and PGK1 was revealed by RIP (**Figure 5G**). Next, we mutated the ac4C site of PGK1, and found that NAT10 knockdown only reduced the luciferase activity of WT-PGK1 but not affected the luciferase activity of MUT-PGK1 (**Figure 5H**). Additionally, NAT10 knockdown decreased the stability of WT-PGK1 mRNA by accelerating its degradation (**Figure 5I**), but not affected the stability of MUT-PGK1 mRNA (**Supplementary Figure S2**), indicating that NAT10 mediated PGK1 mRNA stability through ac4C modification. Notably, emodin downregulated the protein levels of PGK1 in HCT-116 and SW480 cells (**Supplementary Figure S3**), indicating that emodin mediated PGK1 expression through NAT10. Taken together, these results demonstrated that NAT10 knockdown inhibited ac4C modification on PGK1 by suppressing its mRNA stability.

### 3.6 Cell proliferation and glycolysis of colon cancer cells inhibited by NAT10 knockdown is restored by PGK1 overexpression

**Figure 6:**
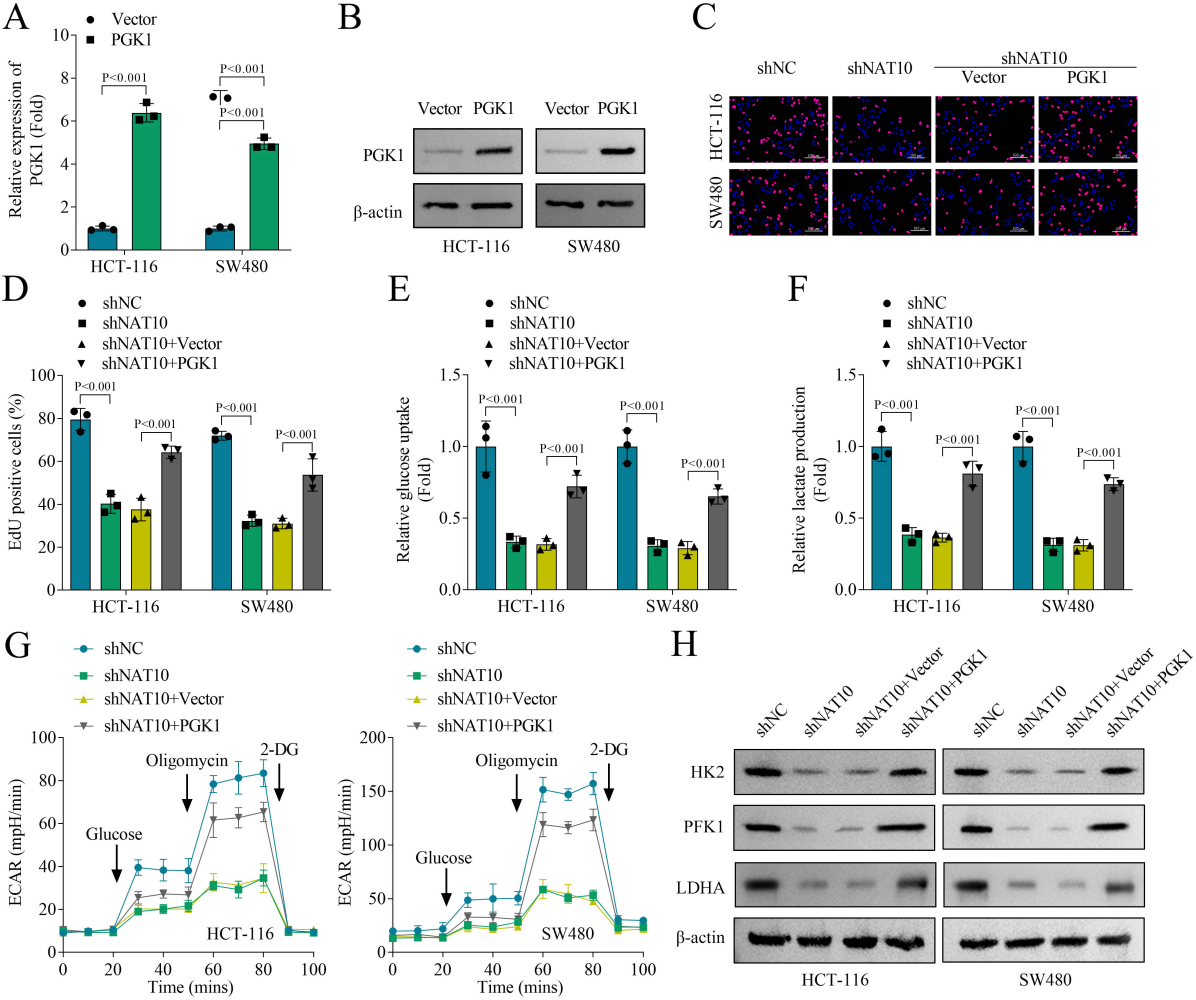
Cell proliferation and glycolysis of colon cancer cells inhibited by NAT10 knockdown was restored by PGK1 overexpression. **(A)** The expression of PGK1 was measured by qPCR. **(B)** The protein levels of PGK1 were detected by western blot. **(C, D)** Cell proliferation of HCT-116 and SW480 cells was evaluated by EdU staining. **(E)** Glucose uptake was evaluated using a glucose uptake assay kit. **(F)** Lactate production was evaluated using a lactate content detection kit. **(G)** Real-time cell metabolism was assessed by measuring ECAR. **(H)** The protein levels of HK2, PFK1 and LDHA were detected by western blot. N = 3 per group. Data analysis was performed using student’s t-test **(A)** or ANOVA **(D–G)**.

Then, we detected the function of PGK1 by transfecting pcDNA3.1-PGK1 into colon cancer cells, and its mRNA expression and protein levels were increased in HCT-116 and SW480 cells (**Figures 6A, B**). EdU staining suggested that NAT10 inhibited cell proliferation of HCT-116 and SW480 cells, which was partially restored by PGK1 overexpression (**Figures 6C, D**). Moreover, glucose uptake, lactate production and ECAR of HCT-116 and SW480 cells inhibited by NAT10 knockdown was partially recovered by PGK1 overexpression (**Figures 6E–G**). Western blot suggested that the protein levels of LDHA and key glycolytic upstream enzymes HK2 and PFK1 in HCT-116 and SW480 cells were decreased by NAT10 knockdown, while this effect was reversed by PGK1 overexpression (**Figure 6H**). In conclusion, these results demonstrated that cell proliferation and glycolysis of colon cancer cells inhibited by NAT10 knockdown was partially restored by PGK1 overexpression.

### 3.7 Emodin inhibits tumor growth and the expression of NAT10 and PGK1 *in vivo*

**Figure 7:**
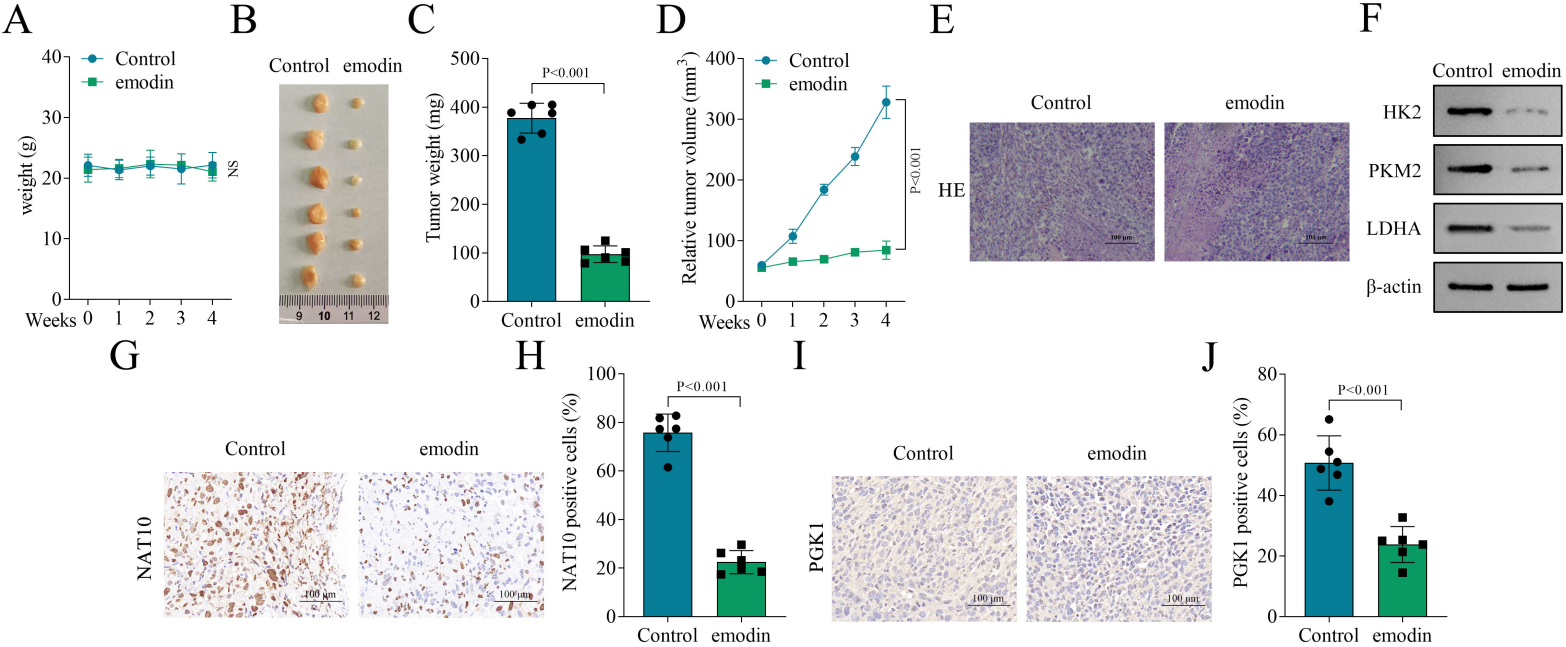
Emodin inhibited tumor growth and the expression of NAT10 and PGK1 *in vivo*. **(A)** The weight of nude mice with emodin treatment or not from week 0-4. **(B–D)** Tumor size, weight and volume of transplanted tumors. **(E)** Pathological change of tumors in the control group and the emodin group was evaluated by HE staining. **(F)** The protein levels of HK2, PKM2 and LDHA were detected by western blot. **(G–J)** The expression of NAT10 and PGK1 of tumors was assessed by IHC staining. NS, no significance. N = 6 per group. Data analysis was performed using student’s t-test.

Finally, the effect of emodin on tumor growth was evaluated by *in vivo* experiments. HCT-116 cells were subcutaneously injected into nude mice. Results showed that emodin not affected the weight of mice, indicating that emodin had no toxicity *in vivo* (**Figure 7A**). Notably, emodin significantly suppressed tumor weight and tumor volume on nude mice (**Figures 7B–D**). HE staining revealed that emodin inhibited the pathological changes of the tumor (**Figure 7E**). Western blot showed that emodin downregulated the protein levels of HK2, PKM2 and LDHA in the nude mice, indicating that emodin inhibited glycolysis in the tumor of the nude mice (**Figure 7F**). Moreover, IHC staining showed that emodin reduced NAT10 and PGK1 expression in the tumors (**Figures 7G–J**). Taken together, these results demonstrated that emodin inhibited tumor growth as well as NAT10 and PGK1 expression *in vivo*.

## 4 Discussion

Emodin is an active ingredient in Chinese medicine derived from plants, which is mainly used in Chinese medicine to treat sore throat, carbuncle, sores and blood stasis (23). Emodin exhibits a diverse range of biological activities, demonstrating potent anti-inflammatory, antioxidant, and antimicrobial effects (24–26). In recent years, the anti-cancer properties of emodin have been extensively studied. For instance, Zou et al. (27) demonstrated that emodin inhibits vascular development and blocks tumor angiogenesis in breast cancer. Emodin has also shown promising antitumor effects in colon cancer, modulating tumor microenvironment and inhibiting colon cancer cell migration and invasion through the regulation of apoptosis and autophagy, as well as the inhibition of epithelial-mesenchymal transition (16, 22, 28, 29). To further investigate the mechanism of emodin in combating colon cancer tumors, colon cancer cells were treated with emodin, and we found that emodin inhibited cell proliferation and glycolysis of colon cancer cells, as well as suppressing tumor growth of colon cancer *in vivo*.

Compared with conventional cells, tumor cells preferentially rely on glycolysis rather than oxidative phosphorylation for energy production, as glycolysis is the primary energy production pathway of tumor cells (30). Inhibition of tumor cell glycolysis has shown great potential in the treatment of cancers. It is reported that FTO inhibited glycolytic metabolism in papillary thyroid cancer thereby abrogating tumor growth (31). Liu et al. (32) demonstrated that sulconazole increases radiosensitivity in esophageal cancer by inhibiting glycolysis. Moreover, Qu et al. (33) confirmed that CLDN6 represses c-MYC-mediated aerobic glycolysis to inhibit proliferation in breast cancer. An increasing number of phytochemicals such as paclitaxel and curcumin, have been confirmed to play an anti-tumor role in gastric, lung and ovary cancers (34, 35). A previous study demonstrated that emodin induces necroptosis and inhibited glycolysis in the renal cancer cells (14). However, whether it can also inhibit tumor cell glycolysis in colon cancer had not been reported previously. In the present study, we demonstrated that with the increase of emodin concentration, the inhibition of proliferation and glycolysis of colon cancer cells was enhanced, especially at 20 μm. Gu et al. (22) reported that emodin inhibited cell migration and invasion in a dose-dependent manner. Both their study and our findings demonstrate the effectiveness of emodin in inhibiting colon cancer development at this dose.

Additionally, our results demonstrated that emodin inhibited ac4C modification and NAT10 expression in colon cancer cells. As a writer of ac4C, NAT10 catalyzes ac4C synthesis on mRNA and mediates the development of multiple cancers. In gastric cancer, NAT10 promotes tumor metastasis by stabilizing ac4C modification on COL5A1 mRNA (36). In colon cancer, NAT10 is highly expressed, and previous studies have revealed that NAT10 promotes colon cancer progression by suppressing ferroptosis (21, 37). Moreover, NAT10 has been shown to contribute to the malignant progression of tumors in gastric cancer, cervical cancer and osteosarcoma by promoting glycolysis (20, 38, 39). However, its role in colon cancer cell glycolysis had not been reported previously. Our results demonstrated that upregulation of NAT10 expression in colon cancer cells partially restored the cell proliferation and glycolysis that were inhibited by emodin. Moreover, PGK1 was identified as a direct regulatory target of NAT10. PGK1 catalyzes the first ATP production step in glycolysis, which is related to tumor cell metabolism and cancer development (40). PGK1 is upregulated in cancers and promotes glycolysis in tumor cells (41, 42). Previous studies have reported that post-translational modification of PGK1 protein stabilize its expression, thereby enhancing cancer glycolysis, including in colon cancer (4, 43). Nevertheless, the regulation of PGK1 mRNA by ac4C modification had not been explored. In this study, we demonstrated that NAT10 stabilized PGK1 mRNA through enhanced ac4C modification on PGK1, thereby promoting glycolysis and cell proliferation in colon cancers.

While our findings provide compelling evidence for the interaction between emodin and NAT10, we acknowledge certain limitations. First, the current study lacks direct functional validation of emodin’s inhibitory effect on NAT10 acetyltransferase activity *in vitro*. Although molecular docking and SRP assays suggest a potential interaction, the absence of enzymatic assays leaves a gap in confirming whether this interaction translates to functional inhibition. Future research should focus on overcoming these challenges by employing advanced biochemical techniques, such as recombinant enzyme purification and kinetic inhibition studies, to rigorously validate the functional relevance of emodin-NAT10 interactions. Another limitation of this study is the reliance on dot blot for assessing ac4C levels on PGK1 transcripts, which provides semi-quantitative data rather than precise occupancy measurements. LC-MS/MS, with its superior quantitative accuracy, would offer a more rigorous assessment of ac4C modification stoichiometry on PGK1 mRNA. Future studies will employ LC-MS/MS to validate and quantify ac4C occupancy on PGK1 transcripts, thereby enhancing the precision of our analysis of ac4C-mediated regulation. Another limitation is the use of fixed concentrations instead of IC50-based doses for functional assays. Although a dose-dependent effect was observed, employing IC50 values would allow for more precise mechanistic interpretation. Future work will utilize cell line-specific IC50 concentrations to improve reproducibility and therapeutic relevance.

Taken together, this study demonstrated that emodin inhibited the tumor growth of colon cancer by suppressing glycolysis in tumor cells through inhibiting NAT10-mediated ac4C modification of PGK1. This may provide a new therapeutic target for colon cancer.

## Data Availability

The raw data supporting the conclusions of this article will be made available by the authors, without undue reservation.

## Supplementary material

Supplementary Figure 1 Effects of NAC and DXM on glucose uptake, lactate production and ECAR in colon cancer cells. **(A)** Glucose uptake was evaluated using a glucose uptake assay kit. **(B)** Lactate production was evaluated using a lactate content detection kit. **(C)** Real-time cell metabolism was assessed by measuring ECAR. N = 3 per group. Data analysis was performed using ANOVA.

Supplementary Figure 2 The stability of PGK1 mRNA with ac4C site mutation. The stability of PGK1 mRNA was measured by qPCR after HCT-116 cells treated with 5 μg/mL actinomycin D for 0, 4, 8, and 12 h. N = 3 per group. Data analysis was performed using ANOVA.

Supplementary Figure 3 The protein levels of PGK1 in HCT-116 and SW480 cells. The protein levels of PGK1 in HCT-116 and SW480 cells were detected by western blot. N = 3 per group.
