# Supplementary figures and images for: Emodin inhibits colon cancer tumor growth by suppressing tumor cell glycolysis through inhibition of NAT10-mediated PGK1 ac4C modification
Source: Front Oncol. 2025 Dec 5;15:1575391. doi: 10.3389/fonc.2025.1575391 (PMC12714669; doi:10.3389/fonc.2025.1575391)

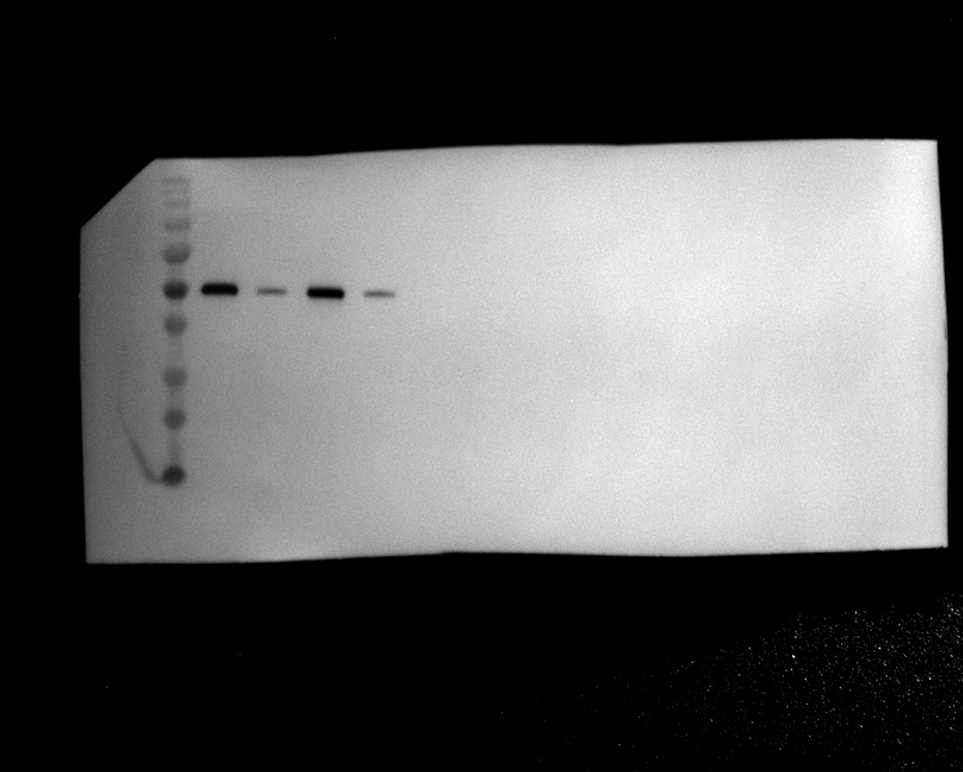

Supplement: Supplementary file 1 [file DataSheet1.zip › Western blot Original Images/Figure 5D Western blot Original Images/1/1-PGK1.tif]

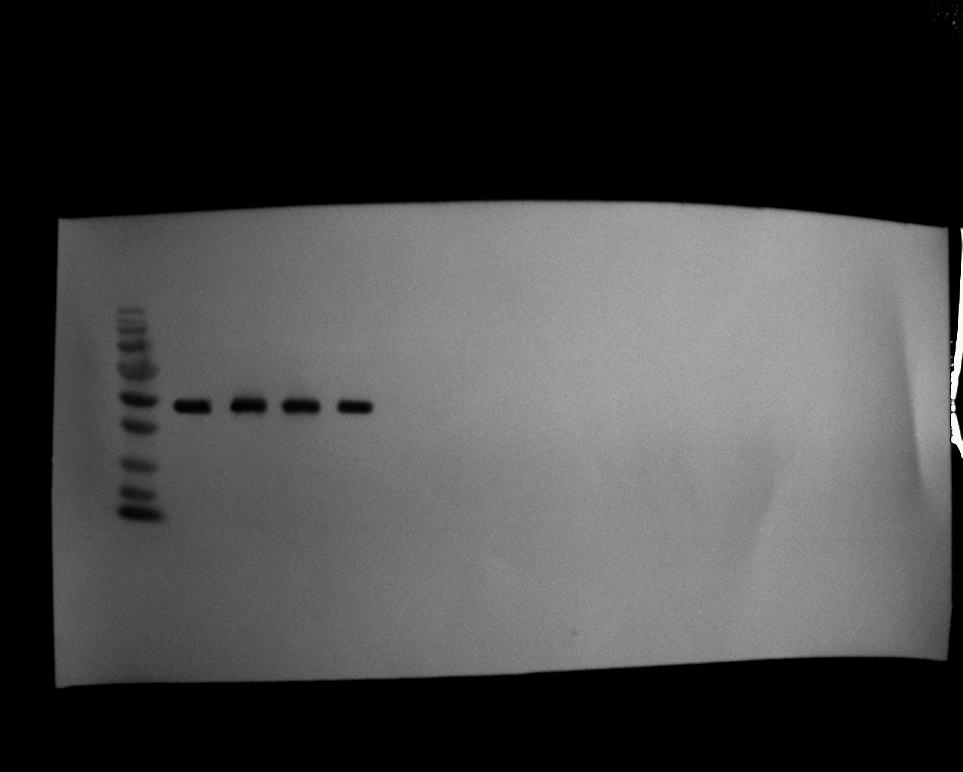

Supplement: Supplementary file 1 [file DataSheet1.zip › Western blot Original Images/Figure 5D Western blot Original Images/1/4-β-actin.tif]

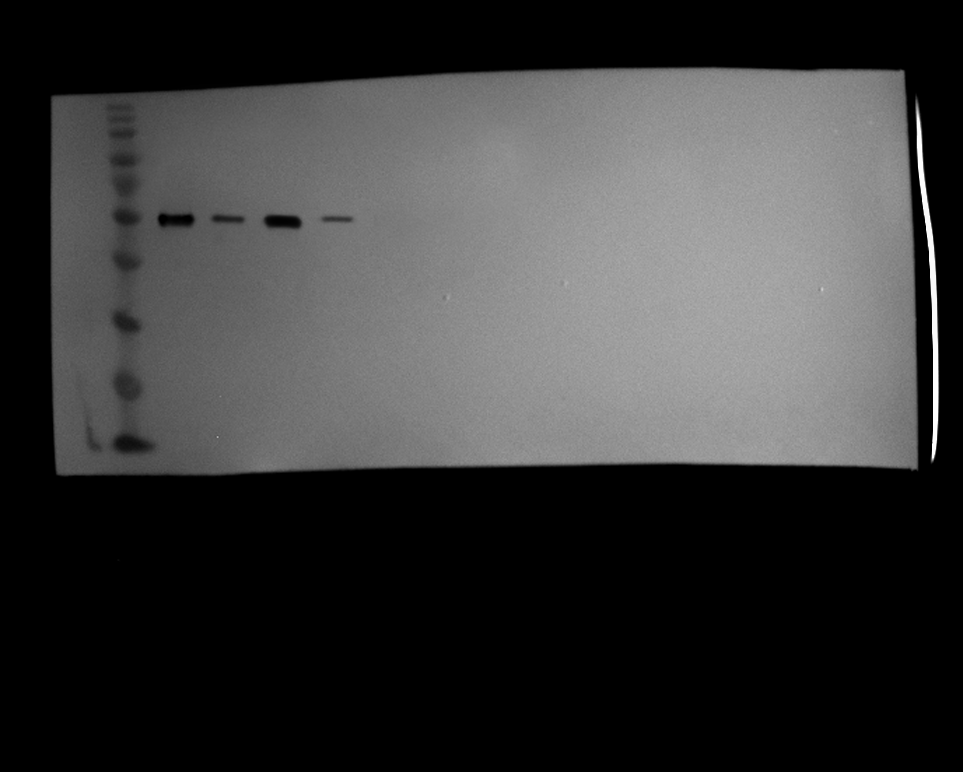

Supplement: Supplementary file 1 [file DataSheet1.zip › Western blot Original Images/Figure 5D Western blot Original Images/2/2-PGK1.tif]

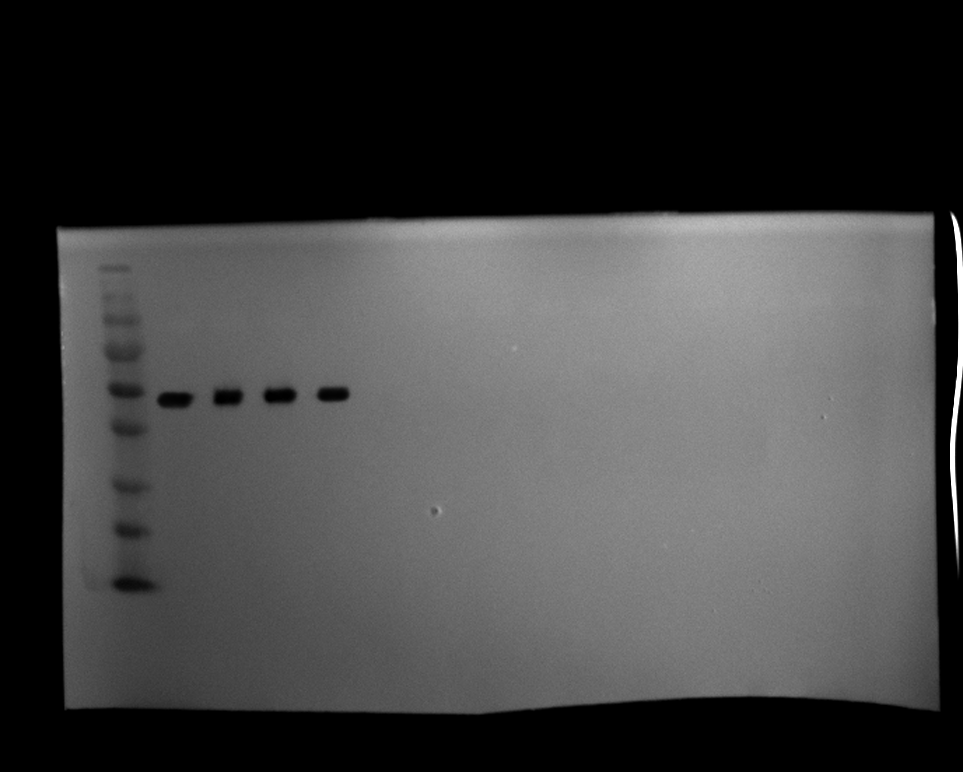

Supplement: Supplementary file 1 [file DataSheet1.zip › Western blot Original Images/Figure 5D Western blot Original Images/2/5-β-actin.tif]

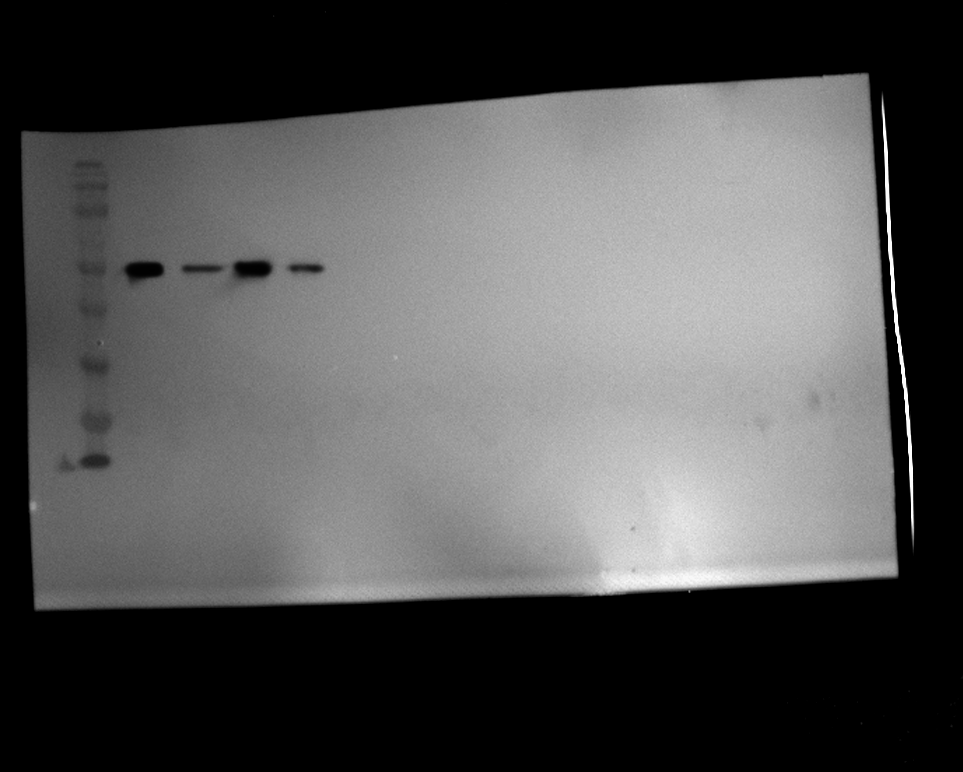

Supplement: Supplementary file 1 [file DataSheet1.zip › Western blot Original Images/Figure 5D Western blot Original Images/3/3-PGK1.tif]

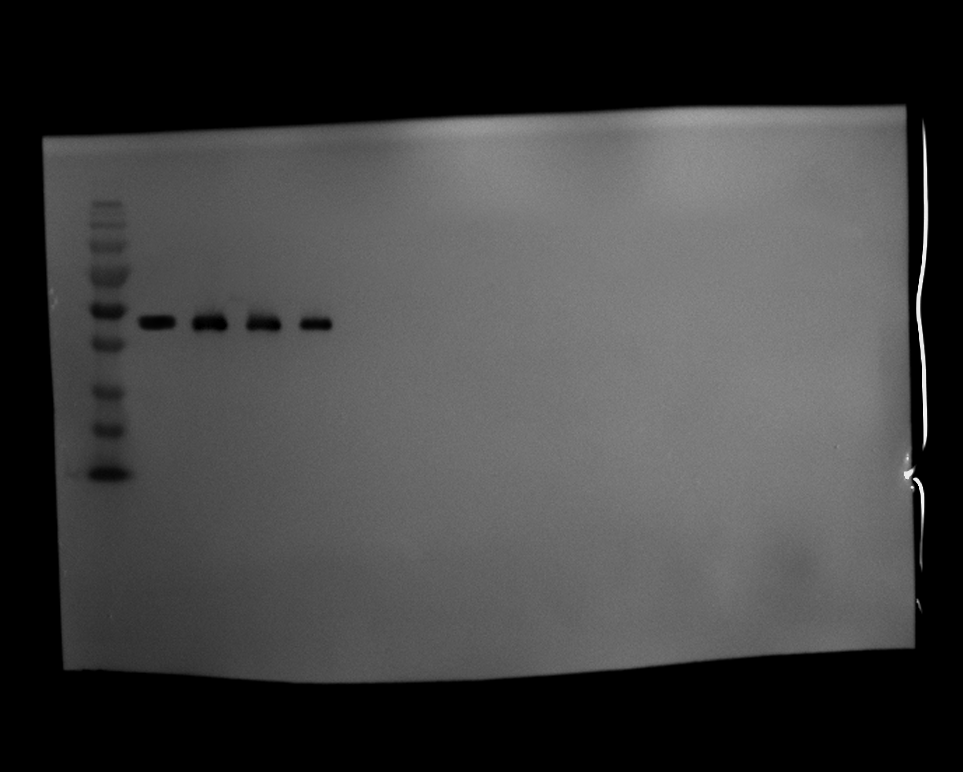

Supplement: Supplementary file 1 [file DataSheet1.zip › Western blot Original Images/Figure 5D Western blot Original Images/3/6-β-actin.tif]

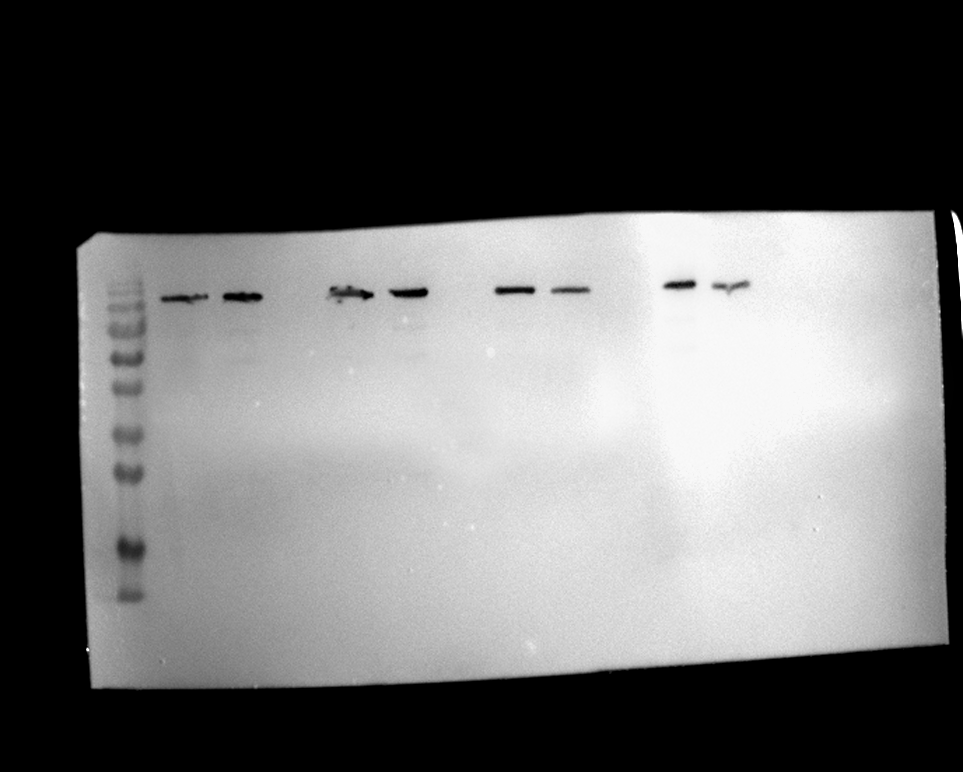

Supplement: Supplementary file 1 [file DataSheet1.zip › Western blot Original Images/Western blot Original Images 1 2/1/4B+5B/1-NAT10.tif]

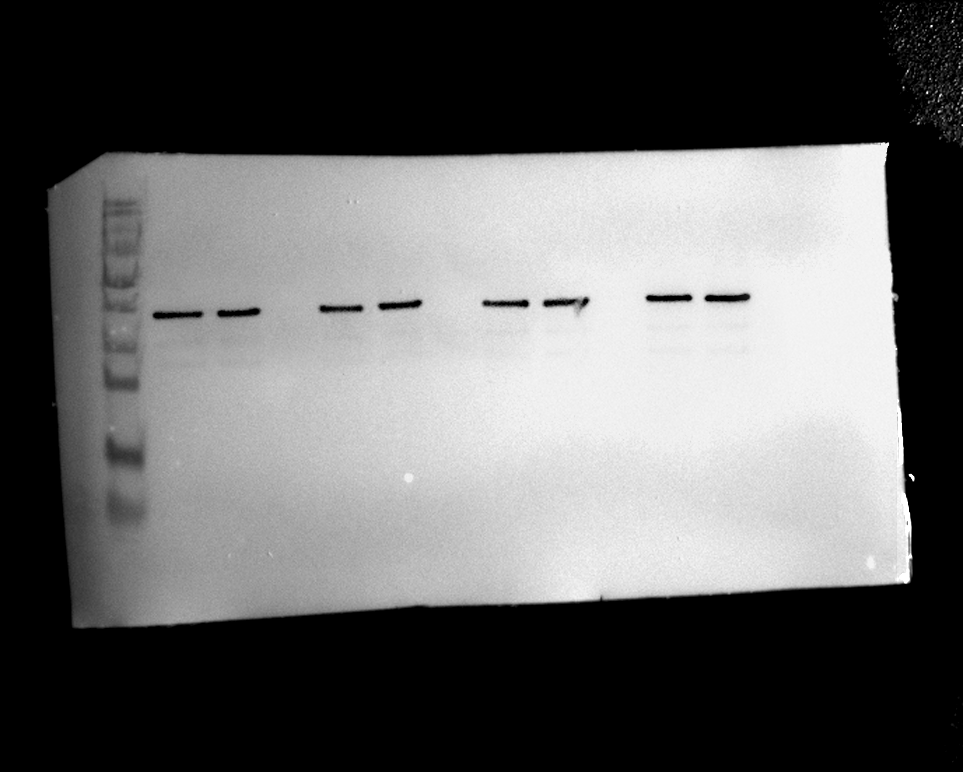

Supplement: Supplementary file 1 [file DataSheet1.zip › Western blot Original Images/Western blot Original Images 1 2/1/4B+5B/2-β-actin.tif]

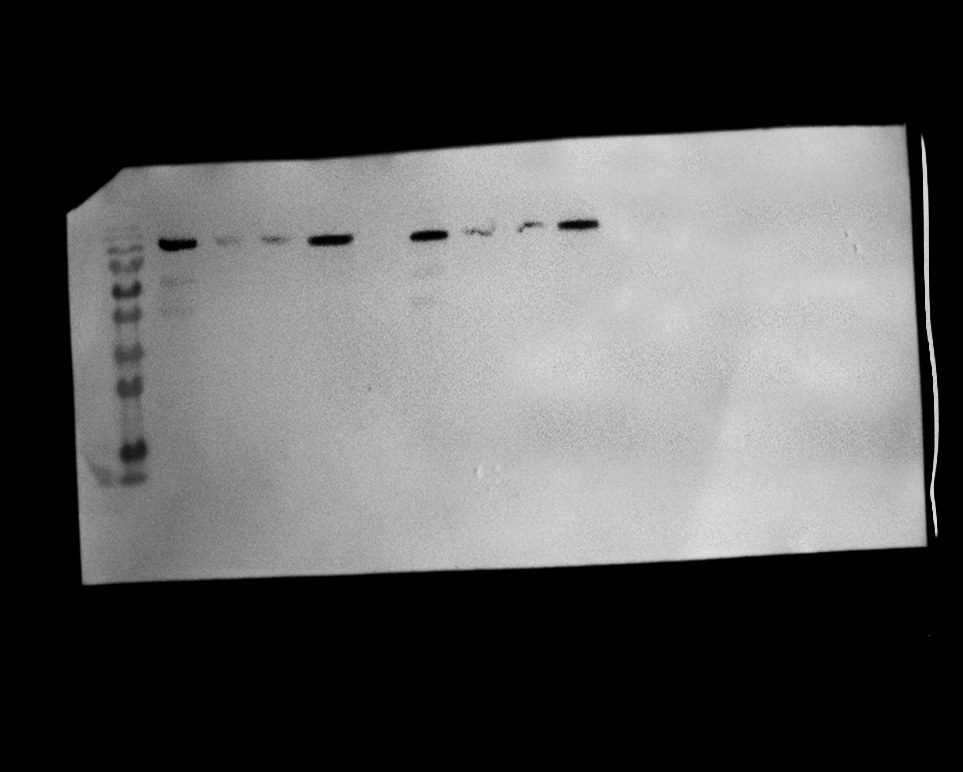

Supplement: Supplementary file 1 [file DataSheet1.zip › Western blot Original Images/Western blot Original Images 1 2/1/4H/1.HK2.tif]

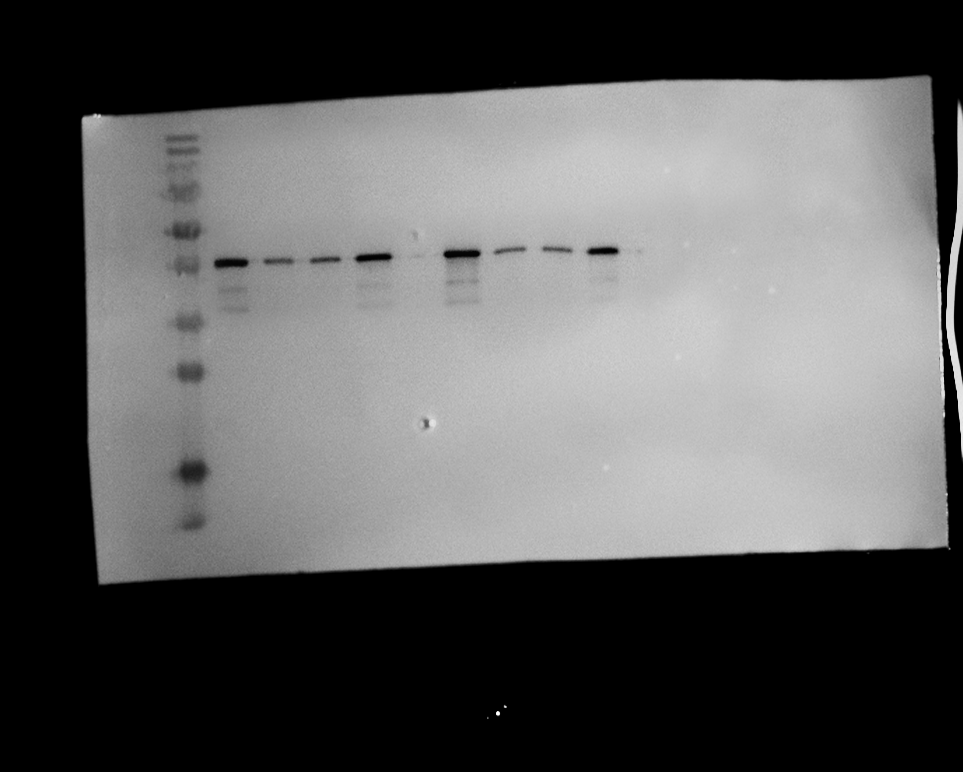

Supplement: Supplementary file 1 [file DataSheet1.zip › Western blot Original Images/Western blot Original Images 1 2/1/4H/2.PGK1.tif]

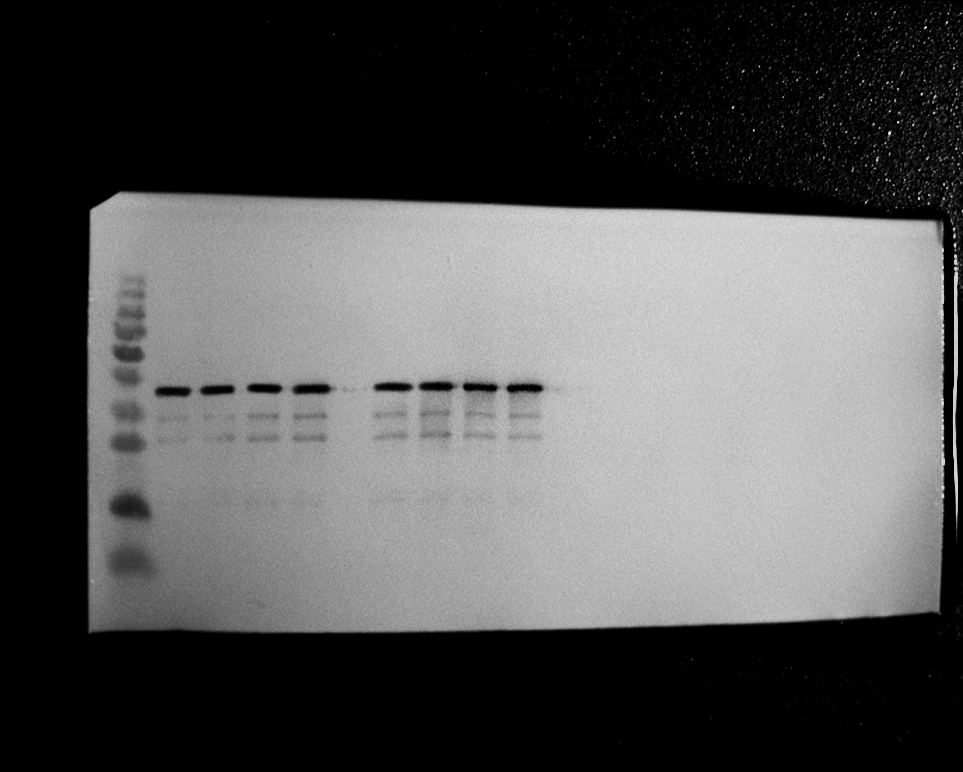

Supplement: Supplementary file 1 [file DataSheet1.zip › Western blot Original Images/Western blot Original Images 1 2/1/4H/3.β-actin.tif]

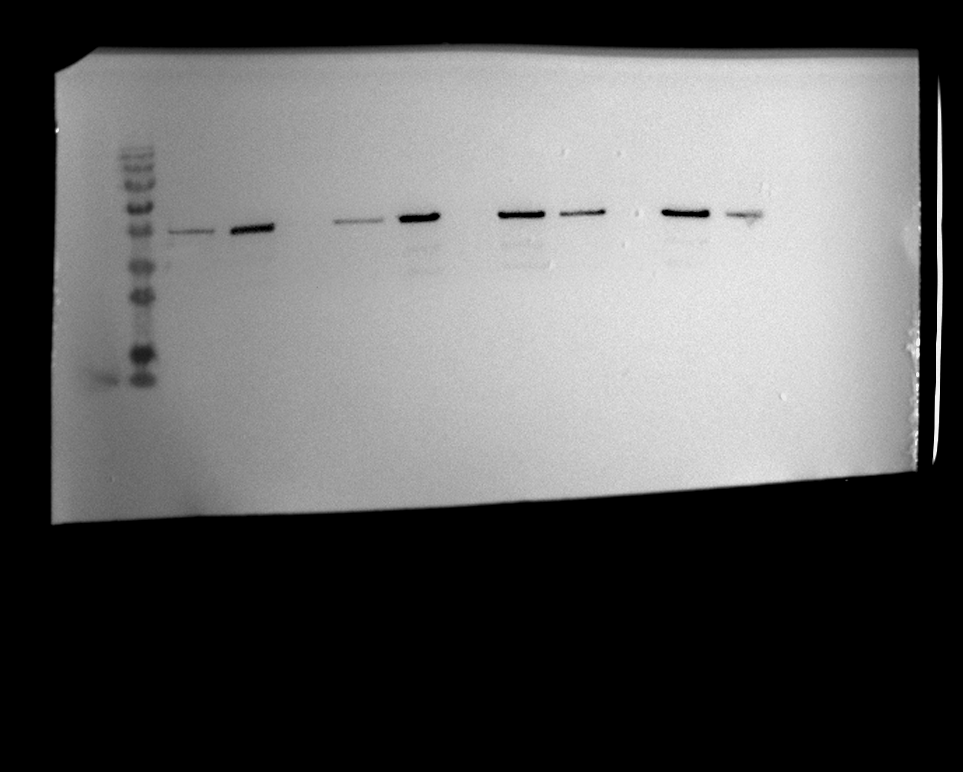

Supplement: Supplementary file 1 [file DataSheet1.zip › Western blot Original Images/Western blot Original Images 1 2/1/6B+S1/1-PGK1.tif]

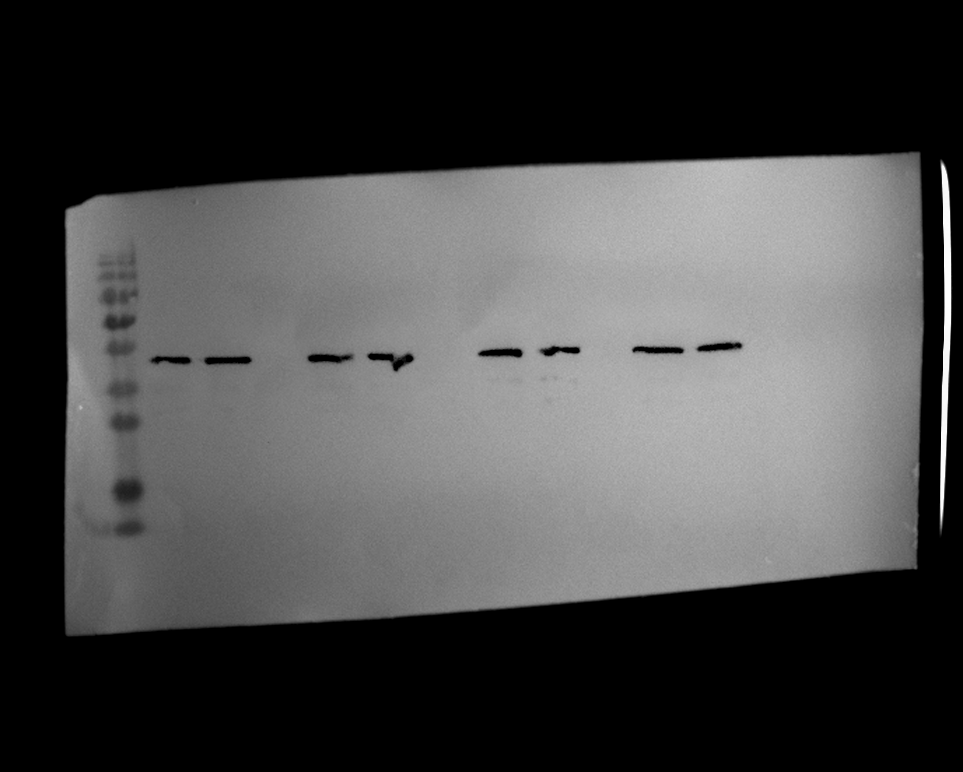

Supplement: Supplementary file 1 [file DataSheet1.zip › Western blot Original Images/Western blot Original Images 1 2/1/6B+S1/2-β-actin.tif]

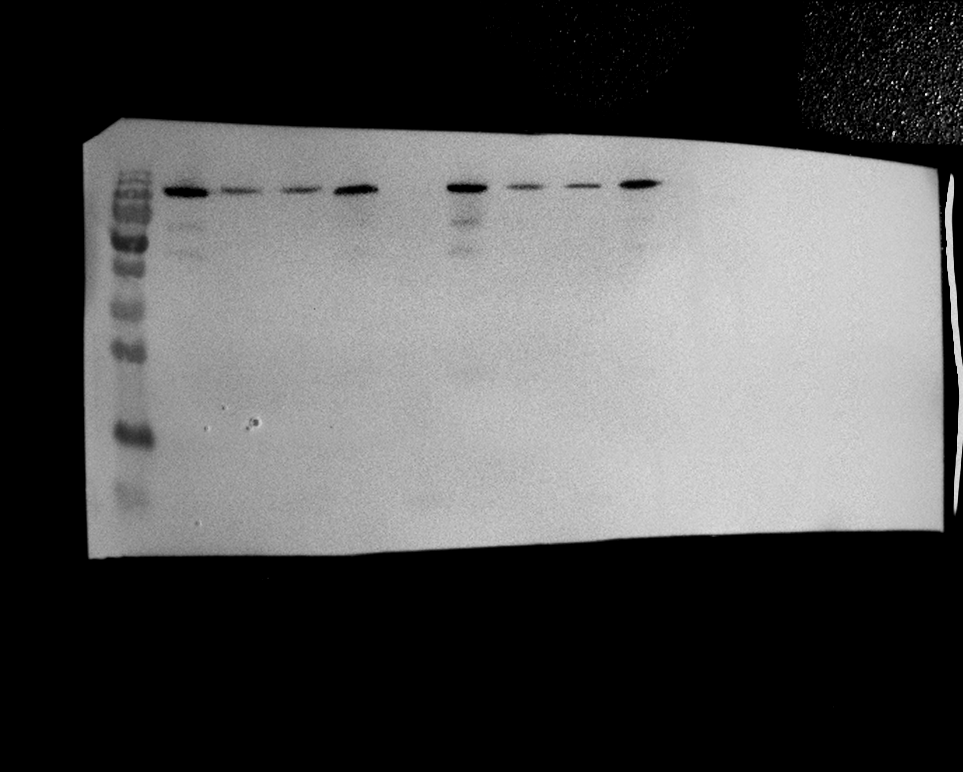

Supplement: Supplementary file 1 [file DataSheet1.zip › Western blot Original Images/Western blot Original Images 1 2/1/6H/1.HK2.tif]

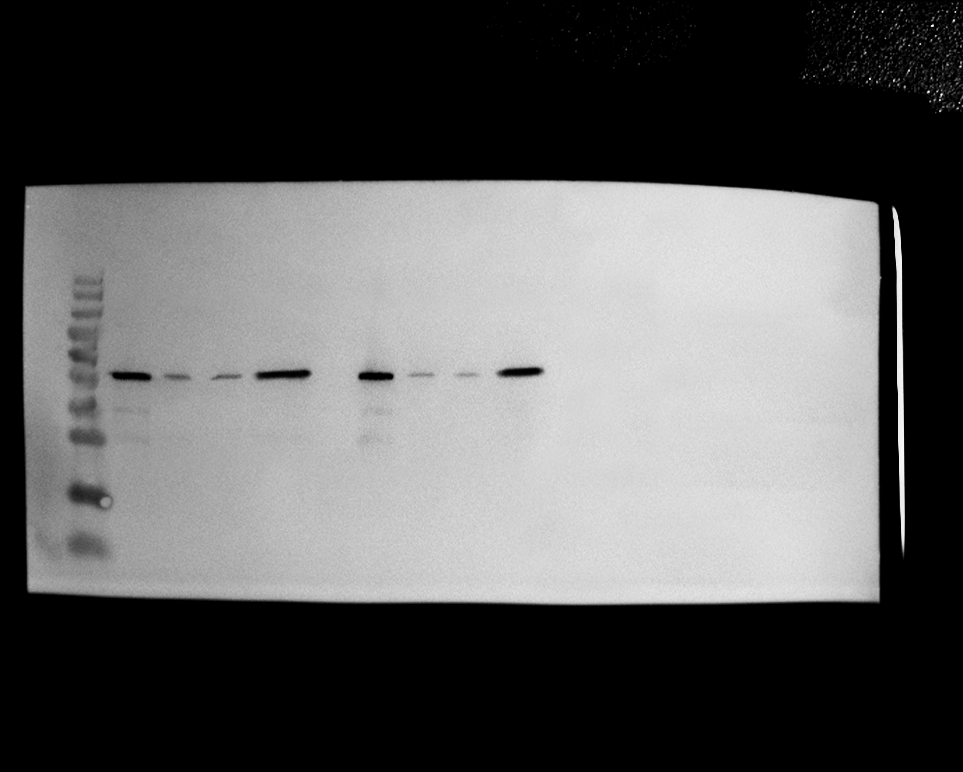

Supplement: Supplementary file 1 [file DataSheet1.zip › Western blot Original Images/Western blot Original Images 1 2/1/6H/2.PGK1.tif]

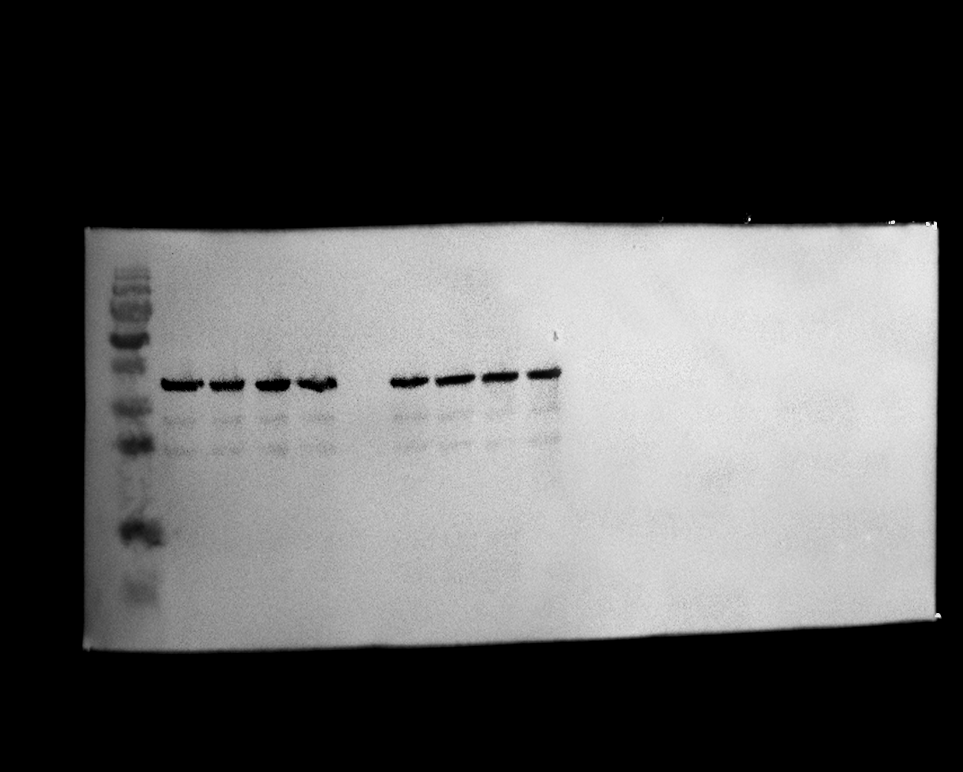

Supplement: Supplementary file 1 [file DataSheet1.zip › Western blot Original Images/Western blot Original Images 1 2/1/6H/3.β-actin.tif]

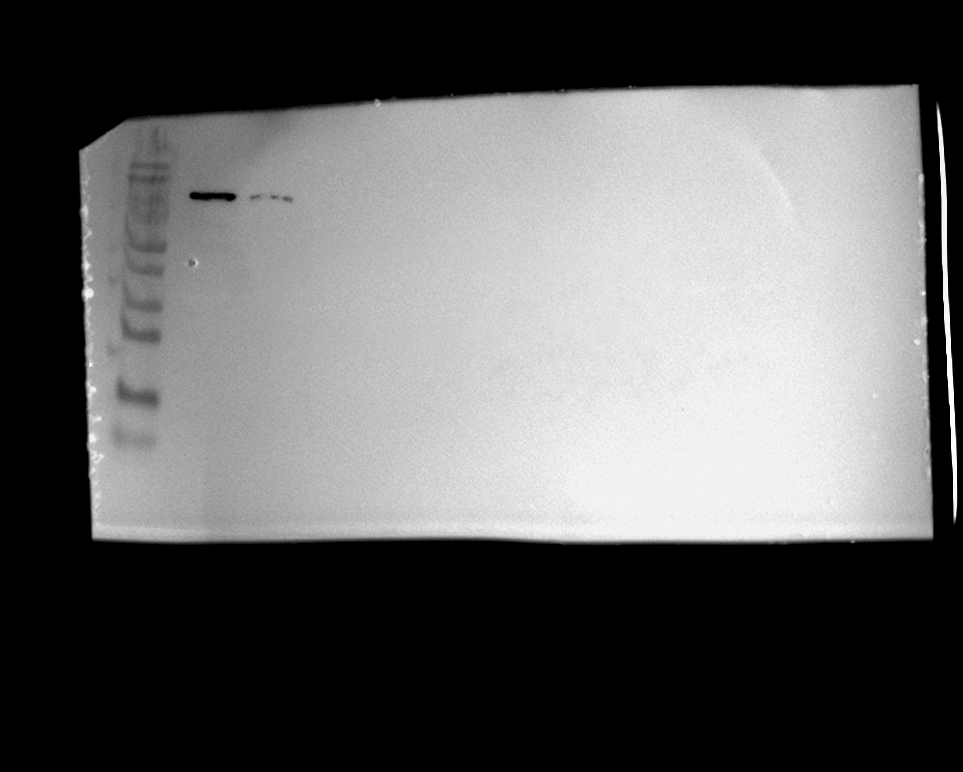

Supplement: Supplementary file 1 [file DataSheet1.zip › Western blot Original Images/Western blot Original Images 1 2/1/7E/1-HK2.tif]

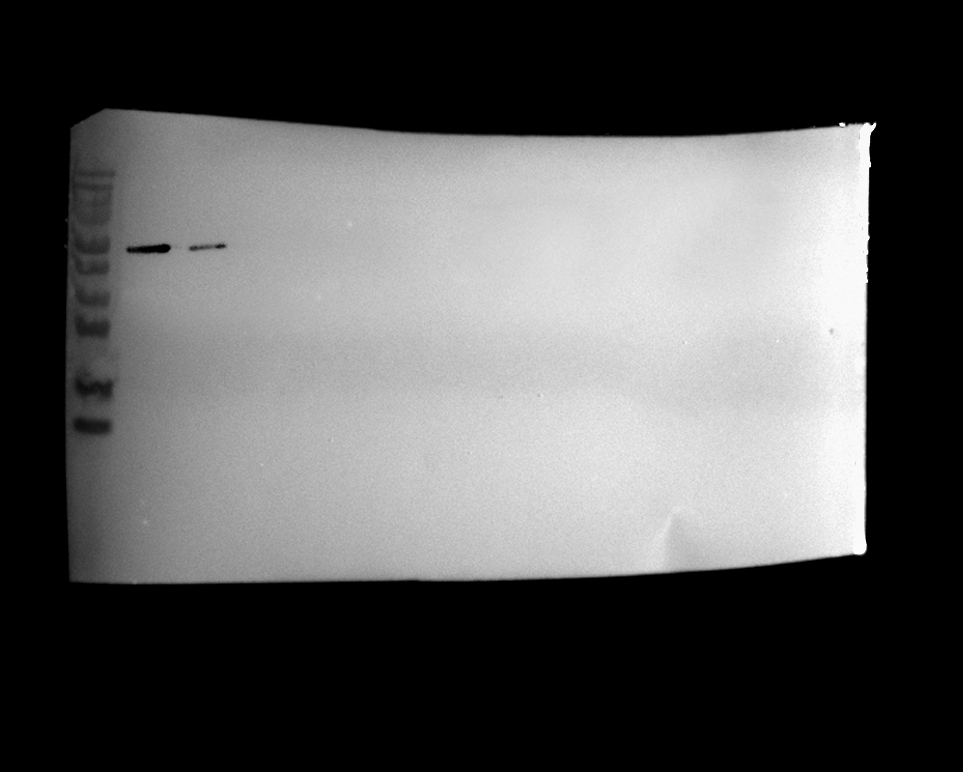

Supplement: Supplementary file 1 [file DataSheet1.zip › Western blot Original Images/Western blot Original Images 1 2/1/7E/2-PKM2.tif]

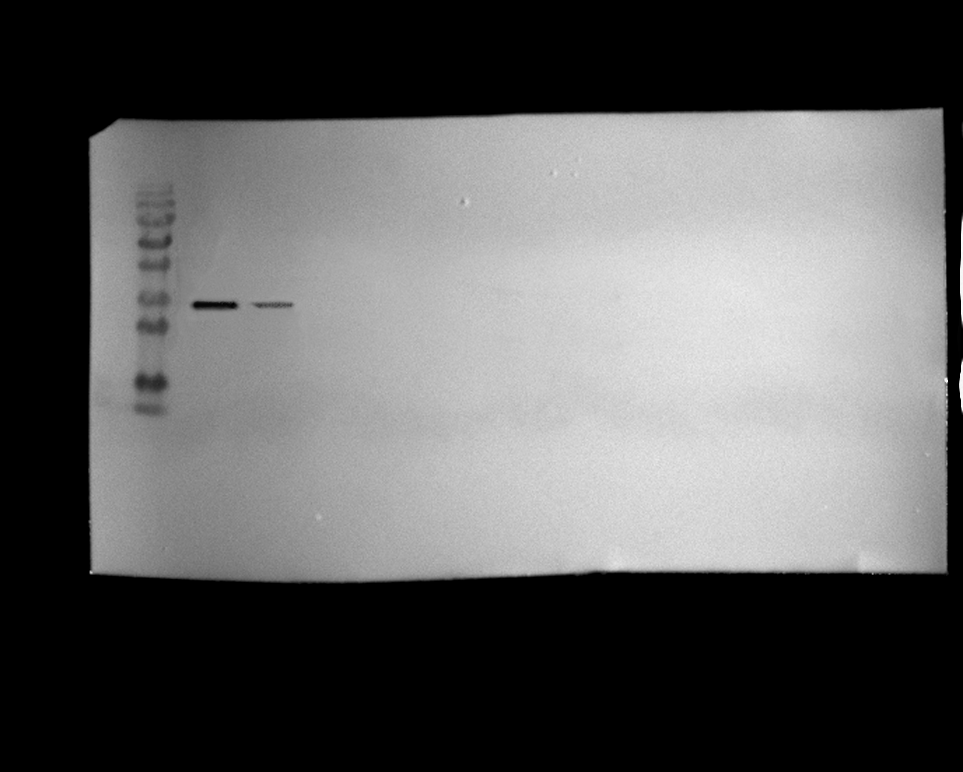

Supplement: Supplementary file 1 [file DataSheet1.zip › Western blot Original Images/Western blot Original Images 1 2/1/7E/3-LDHA.tif]

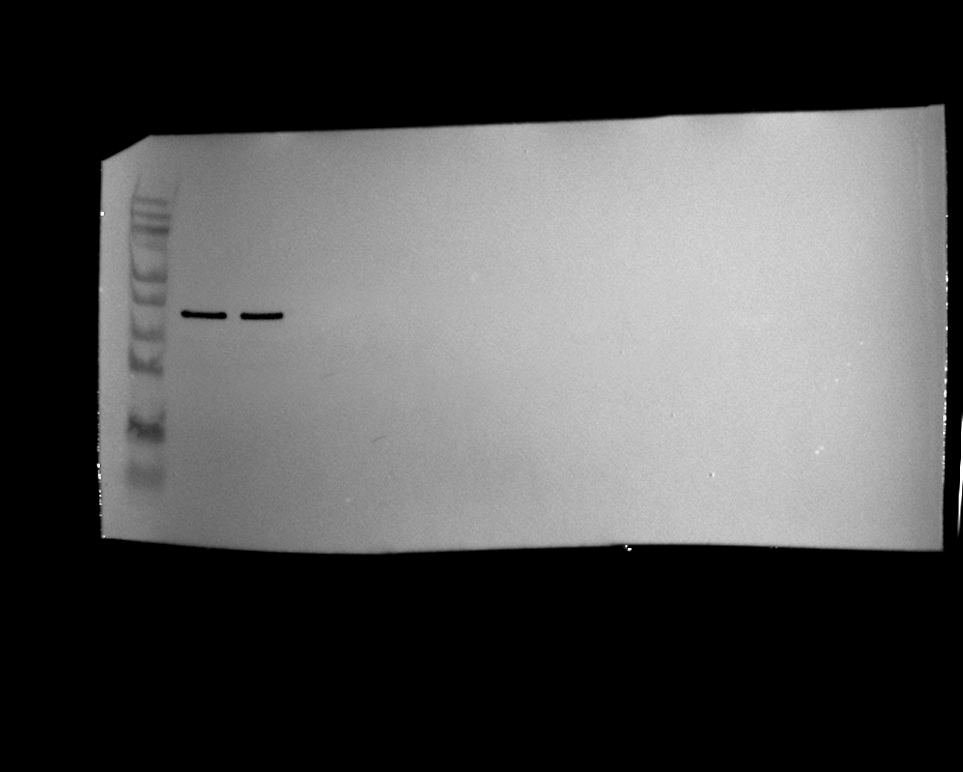

Supplement: Supplementary file 1 [file DataSheet1.zip › Western blot Original Images/Western blot Original Images 1 2/1/7E/4-β-actin.tif]

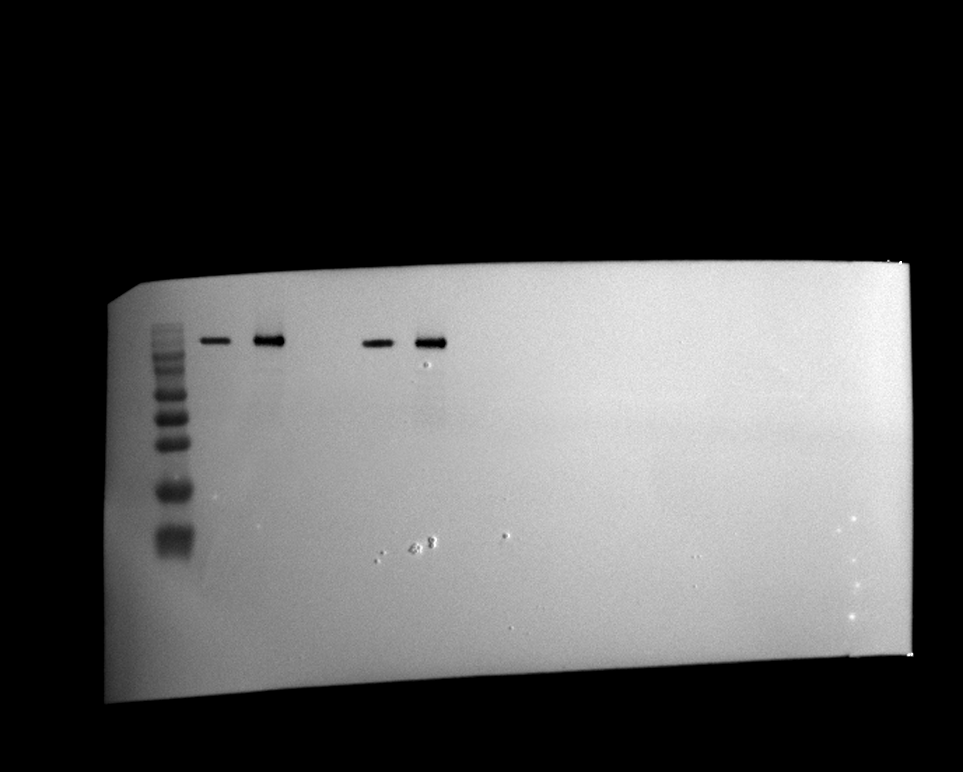

Supplement: Supplementary file 1 [file DataSheet1.zip › Western blot Original Images/Western blot Original Images 1 2/2/4B/1-NAT10.tif]

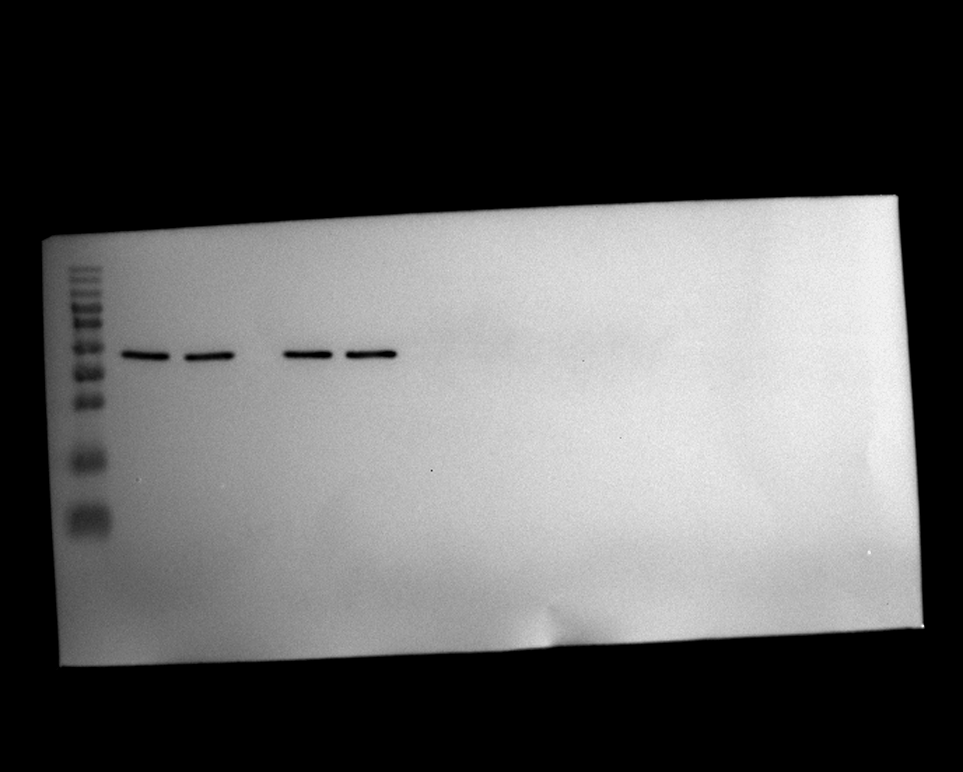

Supplement: Supplementary file 1 [file DataSheet1.zip › Western blot Original Images/Western blot Original Images 1 2/2/4B/2-β-actin.tif]

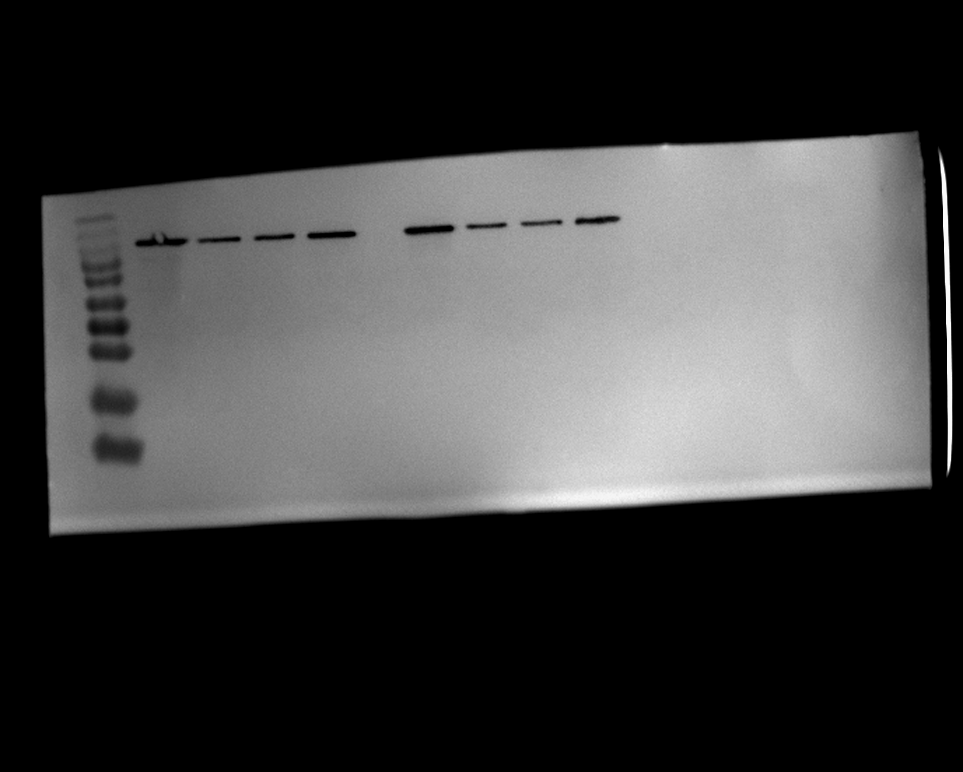

Supplement: Supplementary file 1 [file DataSheet1.zip › Western blot Original Images/Western blot Original Images 1 2/2/4H/1-HK2.tif]

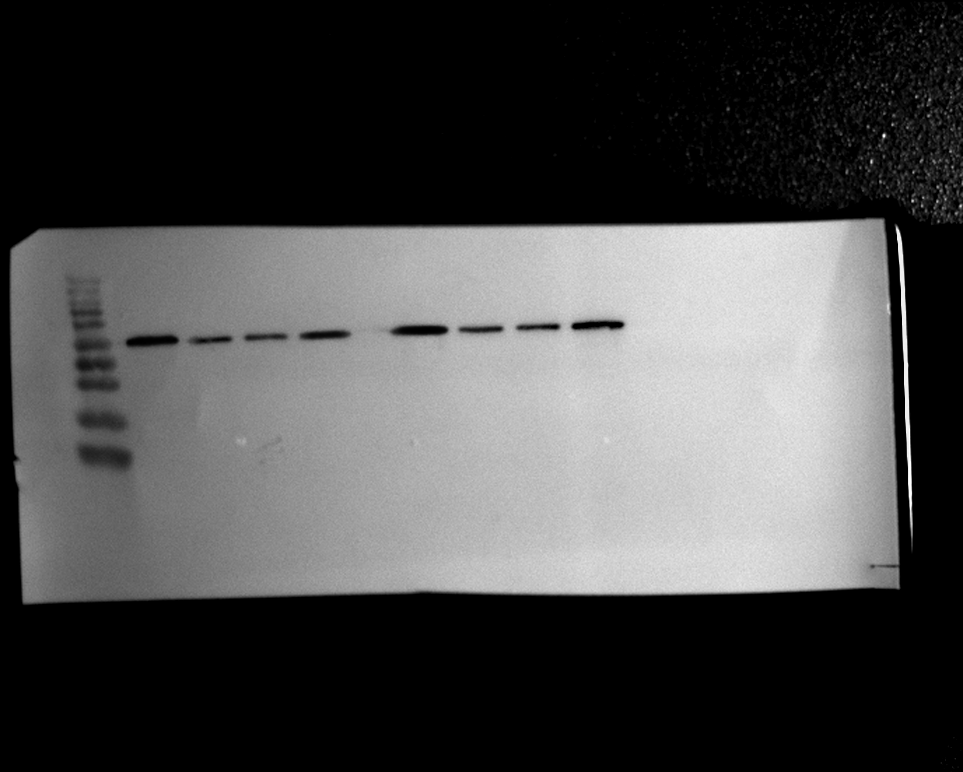

Supplement: Supplementary file 1 [file DataSheet1.zip › Western blot Original Images/Western blot Original Images 1 2/2/4H/2-PGK1.tif]

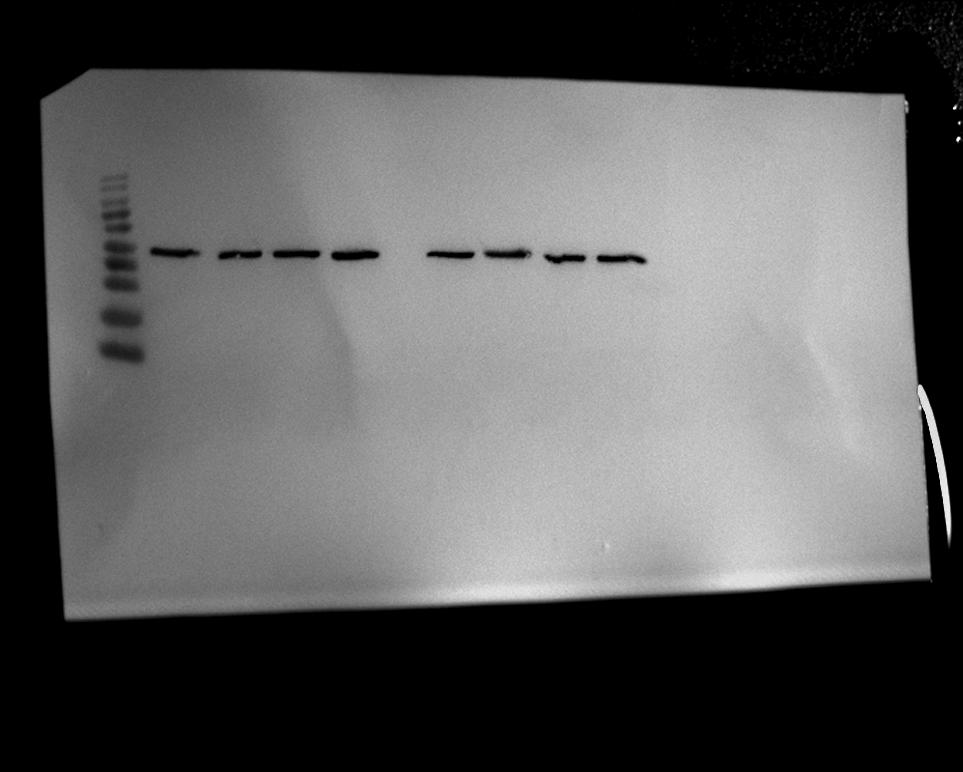

Supplement: Supplementary file 1 [file DataSheet1.zip › Western blot Original Images/Western blot Original Images 1 2/2/4H/3-β-actin.tif]

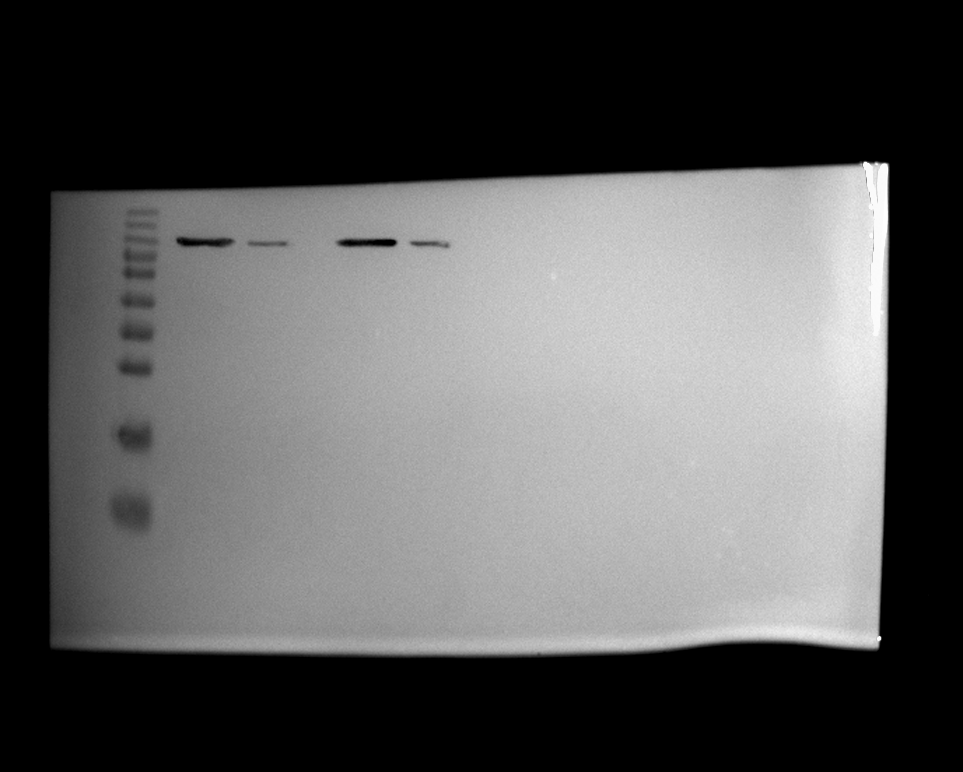

Supplement: Supplementary file 1 [file DataSheet1.zip › Western blot Original Images/Western blot Original Images 1 2/2/5B/1-NAT10.tif]

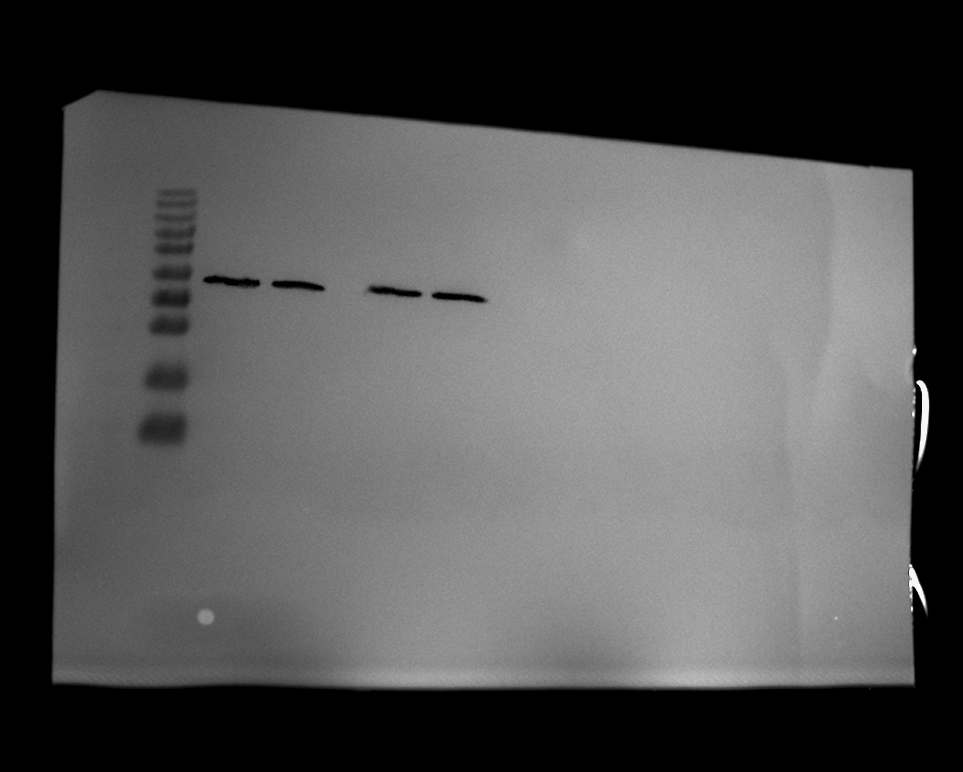

Supplement: Supplementary file 1 [file DataSheet1.zip › Western blot Original Images/Western blot Original Images 1 2/2/5B/2-β-actin.tif]

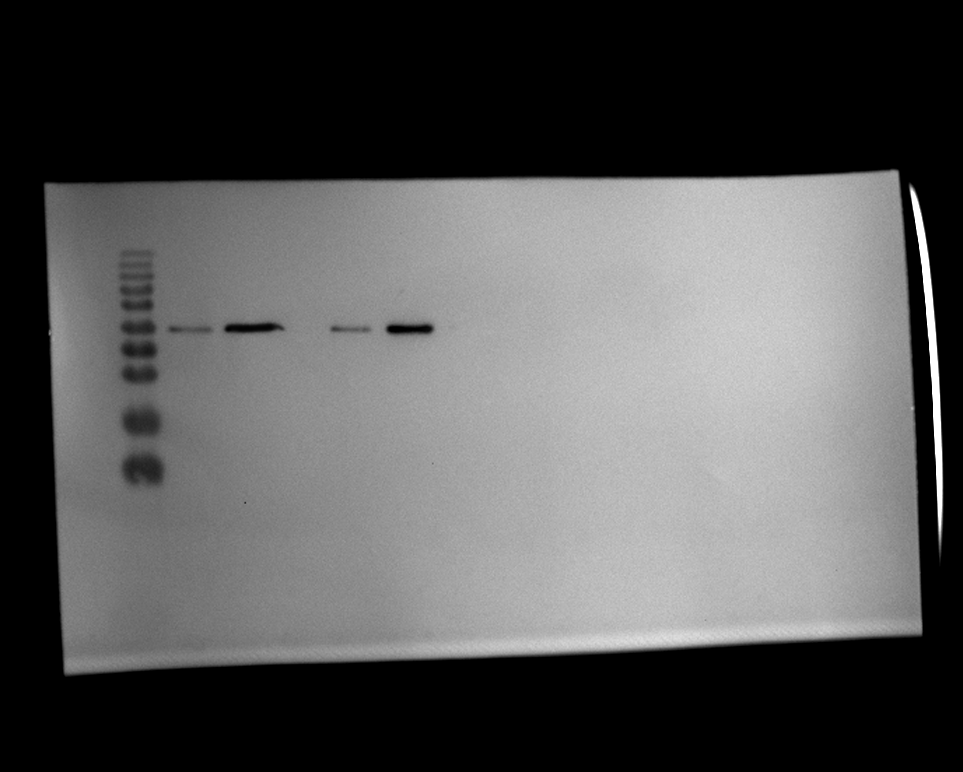

Supplement: Supplementary file 1 [file DataSheet1.zip › Western blot Original Images/Western blot Original Images 1 2/2/6B/1-PGK1.tif]

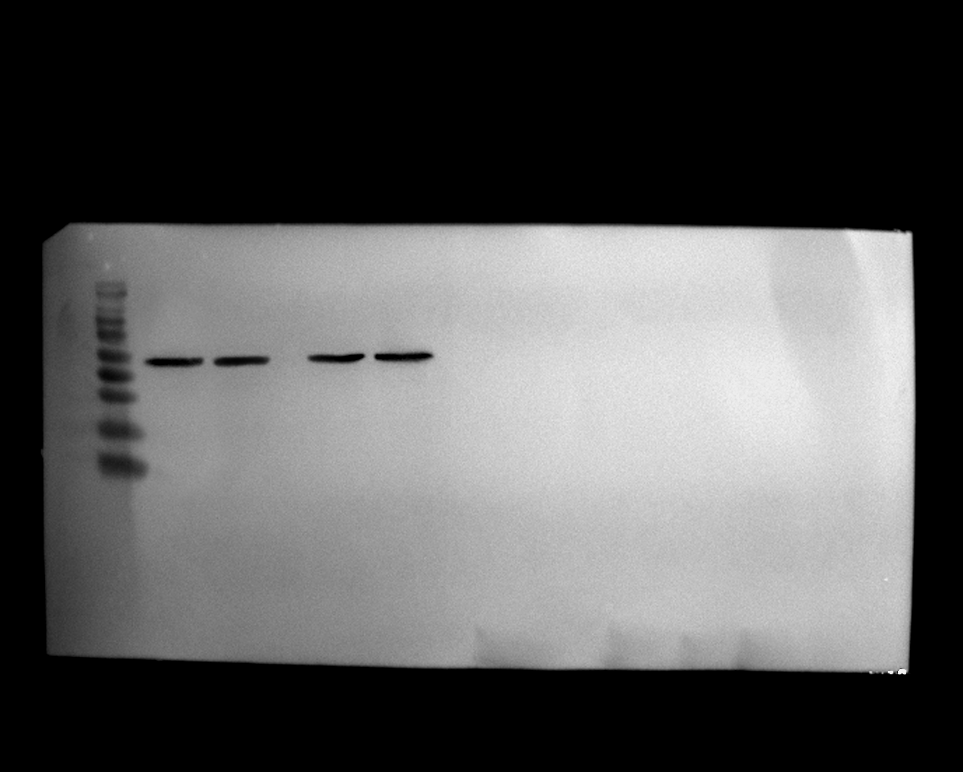

Supplement: Supplementary file 1 [file DataSheet1.zip › Western blot Original Images/Western blot Original Images 1 2/2/6B/2-β-actin.tif]

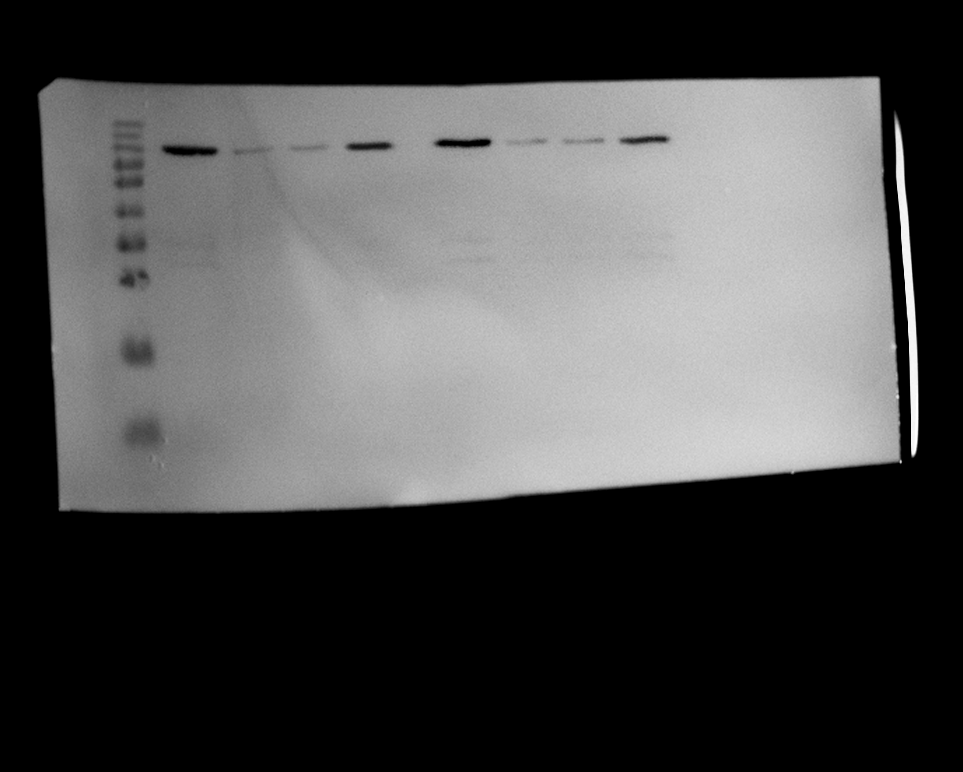

Supplement: Supplementary file 1 [file DataSheet1.zip › Western blot Original Images/Western blot Original Images 1 2/2/6H/1-HK2.tif]

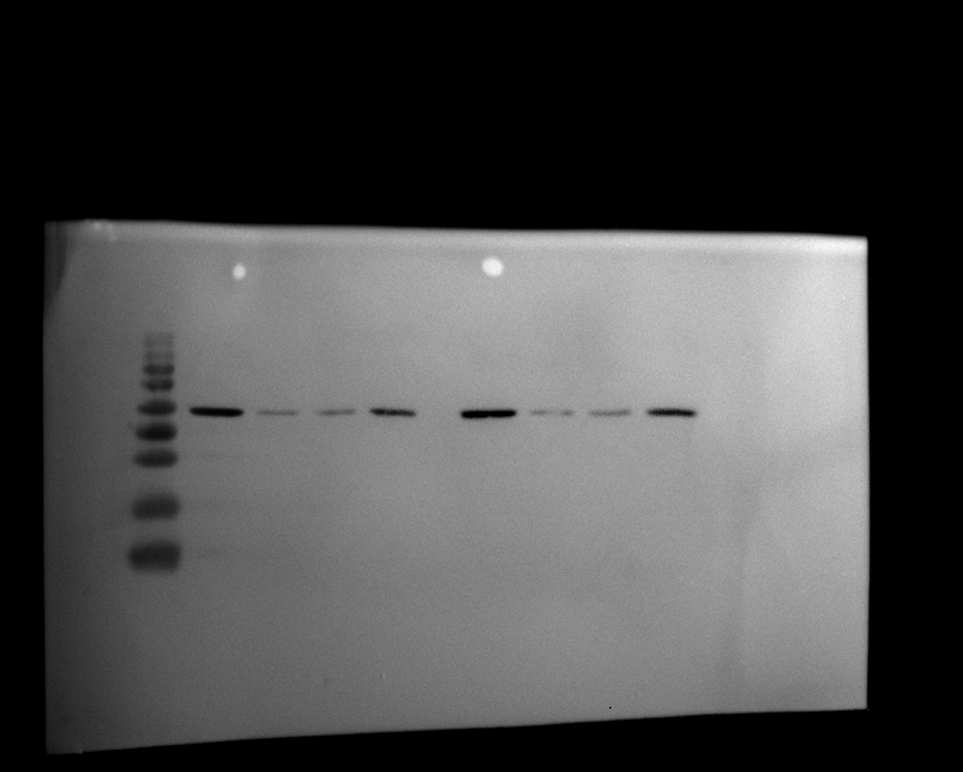

Supplement: Supplementary file 1 [file DataSheet1.zip › Western blot Original Images/Western blot Original Images 1 2/2/6H/2-PGK1.tif]

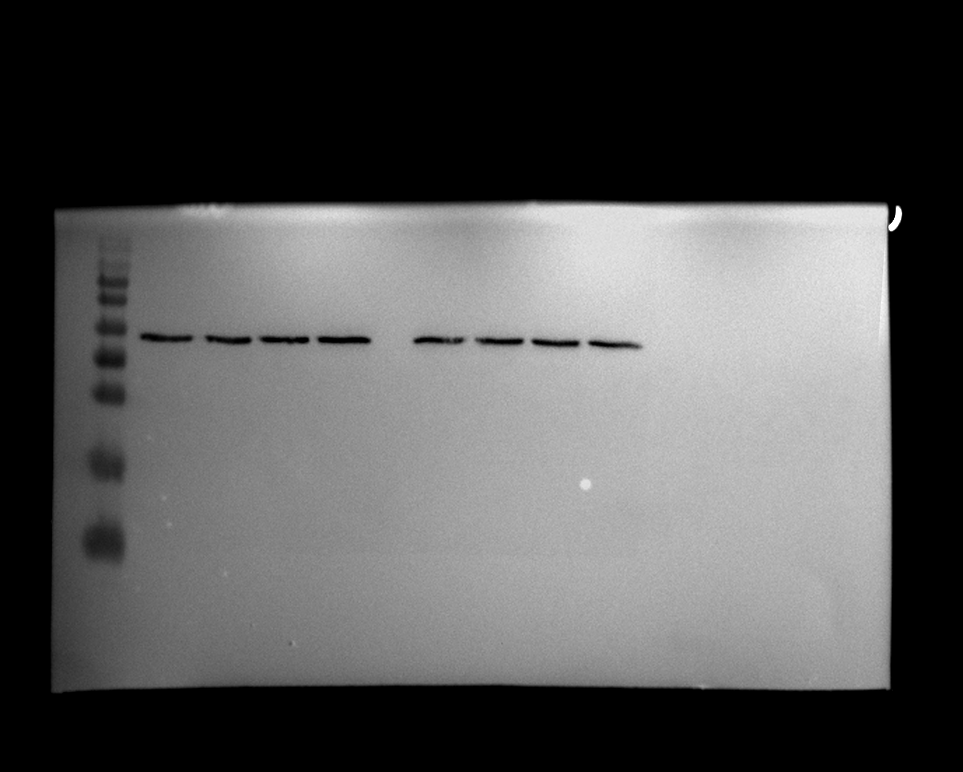

Supplement: Supplementary file 1 [file DataSheet1.zip › Western blot Original Images/Western blot Original Images 1 2/2/6H/3-β-actin.tif]

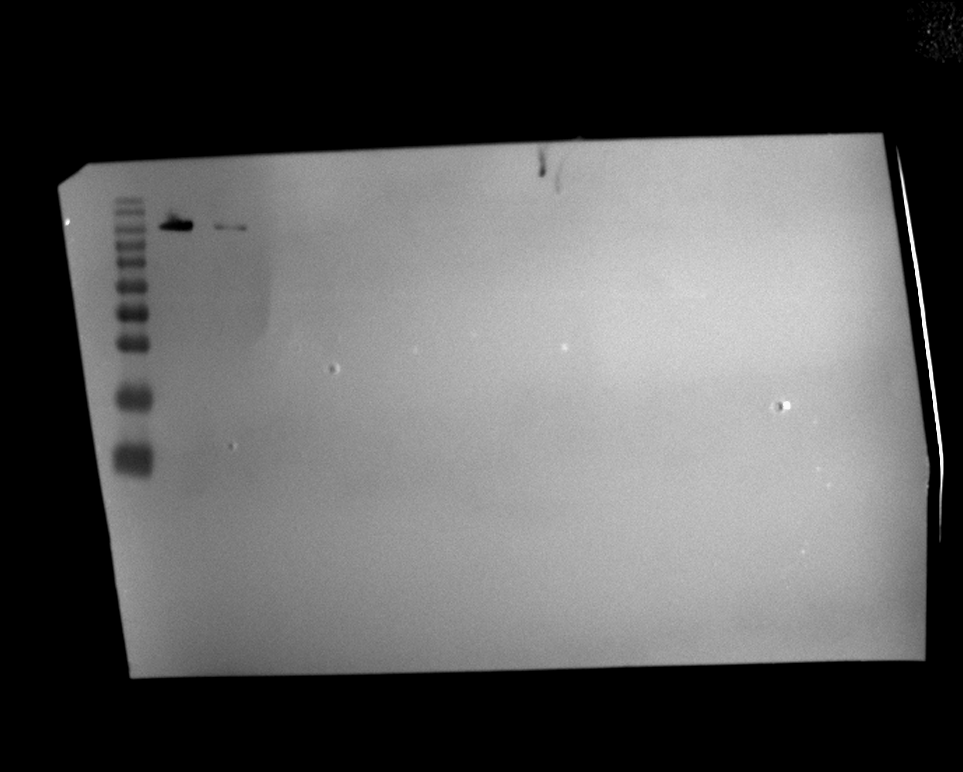

Supplement: Supplementary file 1 [file DataSheet1.zip › Western blot Original Images/Western blot Original Images 1 2/2/7E/1-HK2.tif]

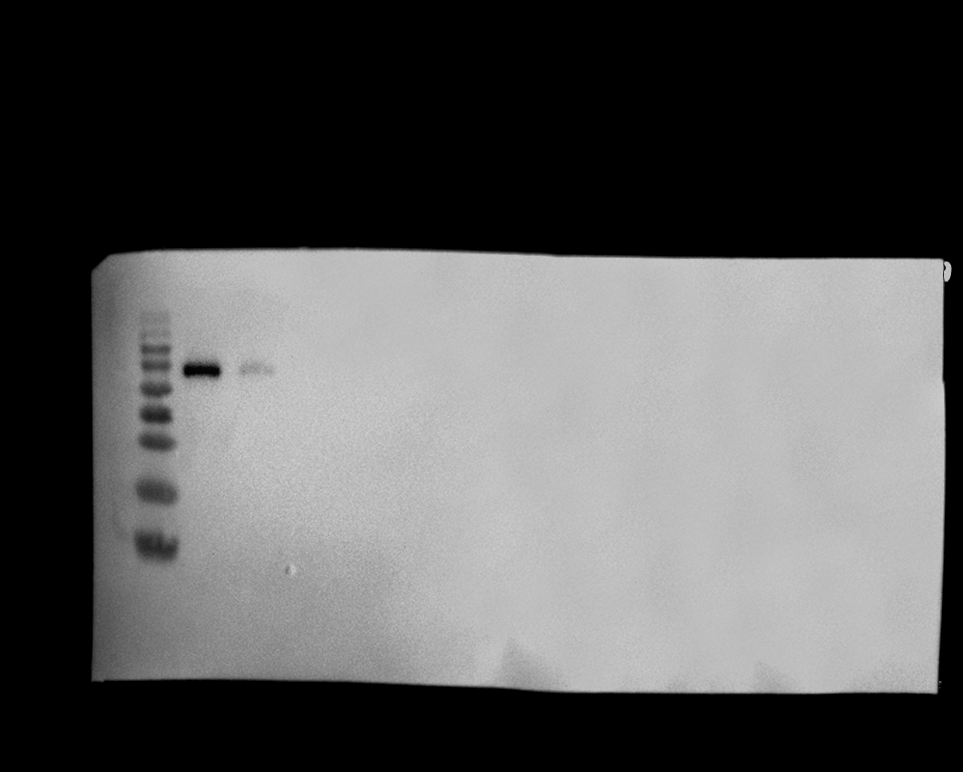

Supplement: Supplementary file 1 [file DataSheet1.zip › Western blot Original Images/Western blot Original Images 1 2/2/7E/2-PKM2.tif]

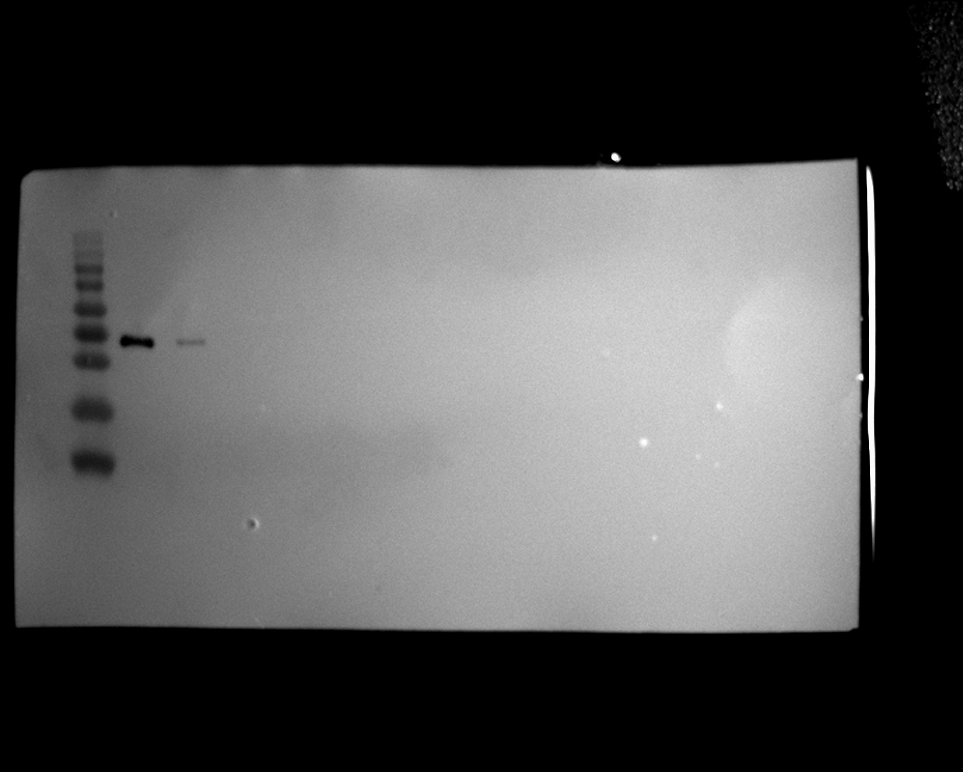

Supplement: Supplementary file 1 [file DataSheet1.zip › Western blot Original Images/Western blot Original Images 1 2/2/7E/3-LDHA.tif]

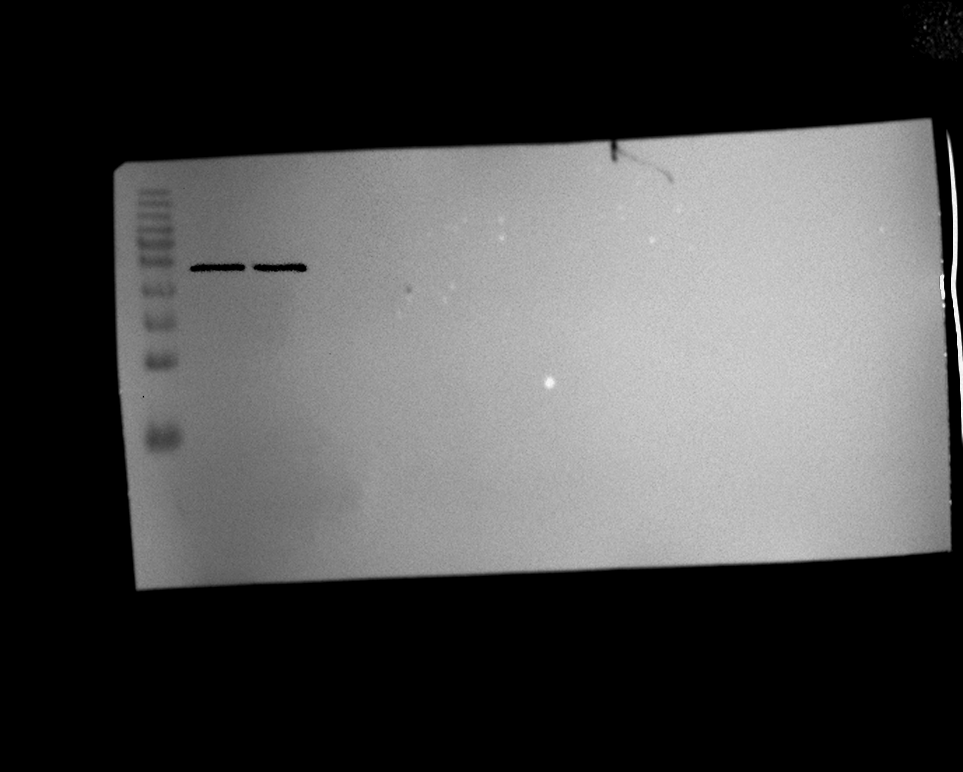

Supplement: Supplementary file 1 [file DataSheet1.zip › Western blot Original Images/Western blot Original Images 1 2/2/7E/4-β-actin.tif]

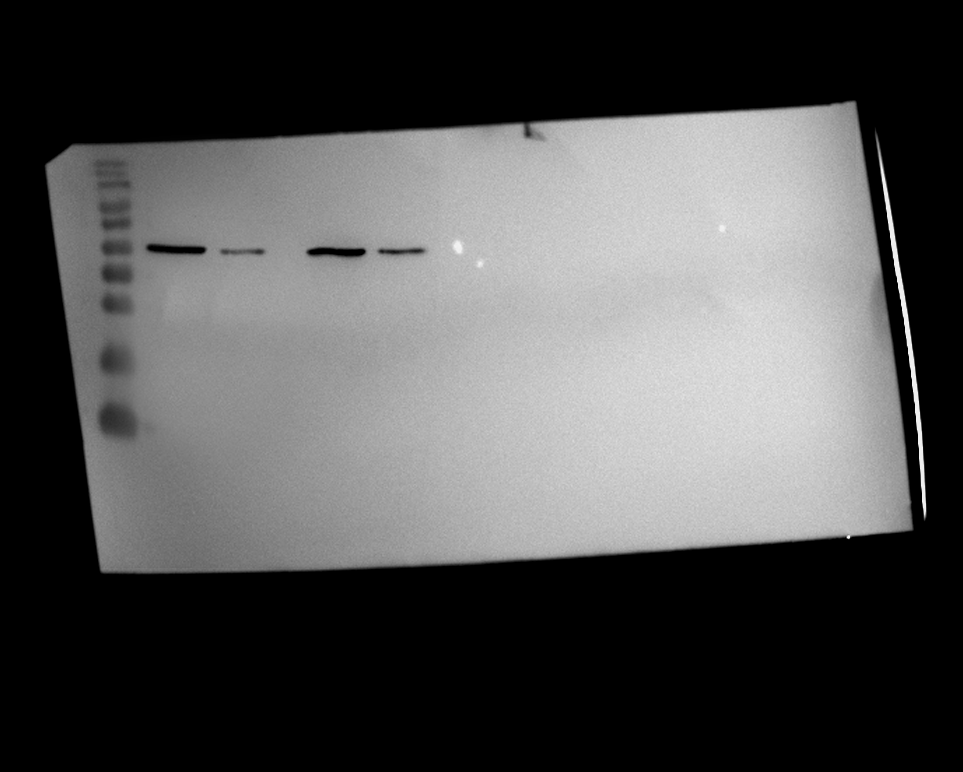

Supplement: Supplementary file 1 [file DataSheet1.zip › Western blot Original Images/Western blot Original Images 1 2/2/S1/1-PGK1.tif]

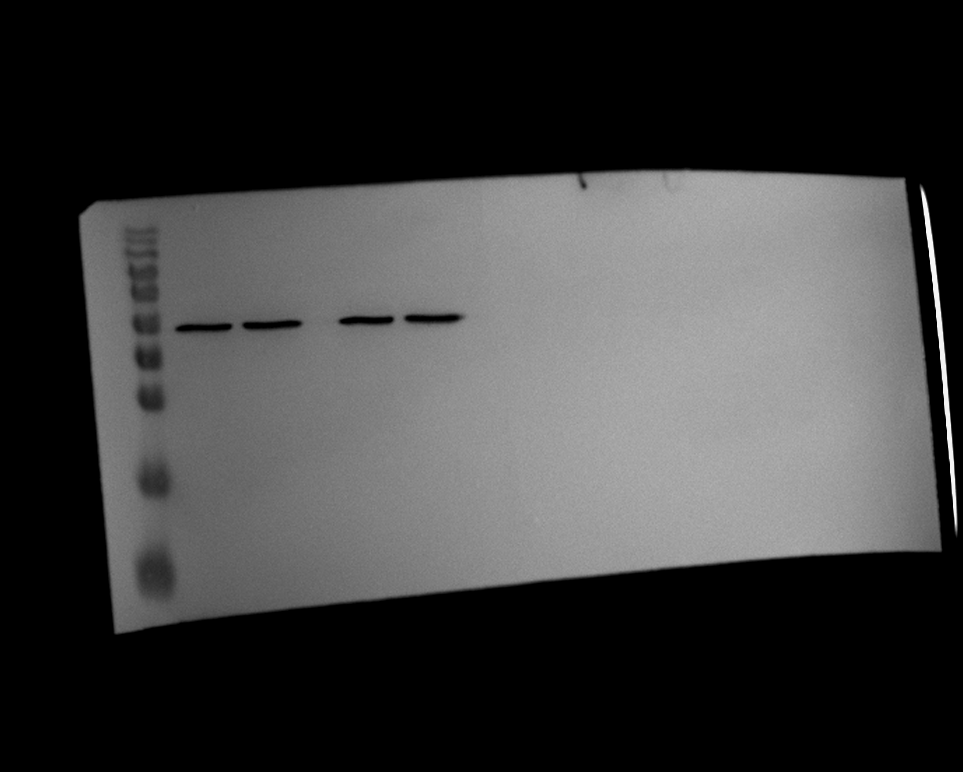

Supplement: Supplementary file 1 [file DataSheet1.zip › Western blot Original Images/Western blot Original Images 1 2/2/S1/2-β-actin.tif]

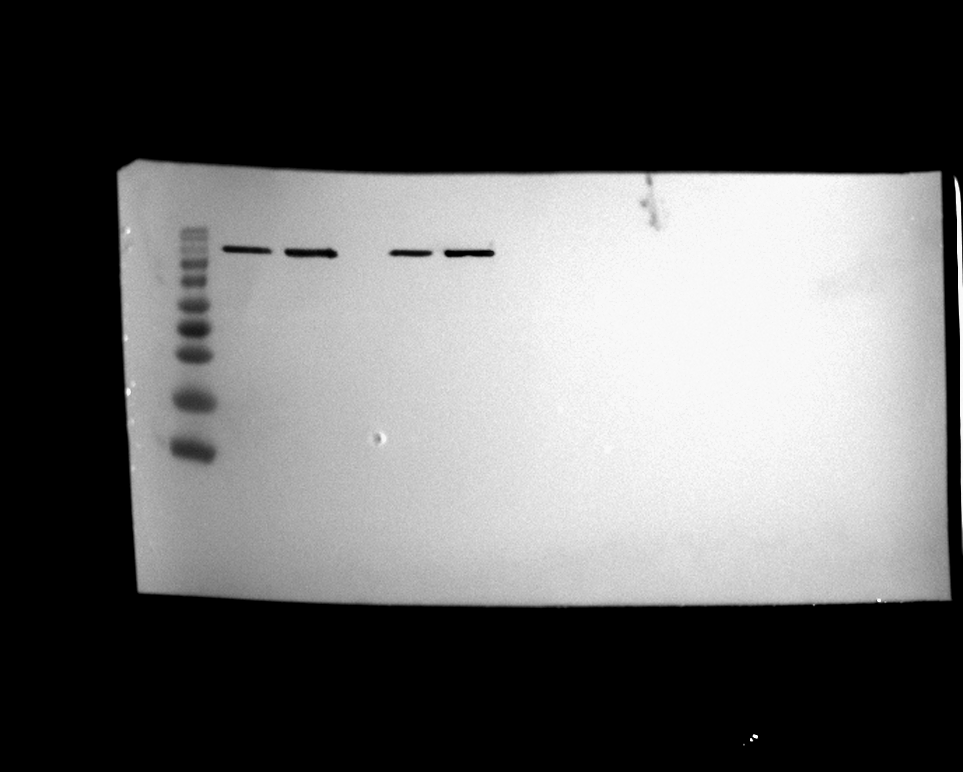

Supplement: Supplementary file 1 [file DataSheet1.zip › Western blot Original Images/Western blot Original Images 3/3/4B/1-NAT10.tif]

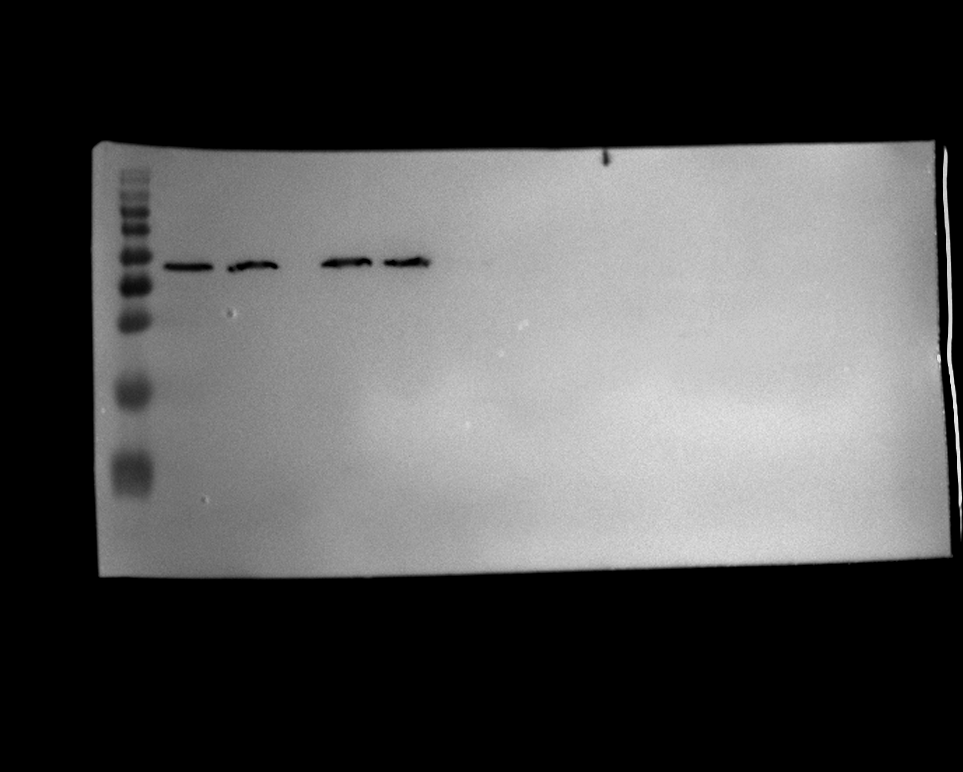

Supplement: Supplementary file 1 [file DataSheet1.zip › Western blot Original Images/Western blot Original Images 3/3/4B/2-β-actin.tif]

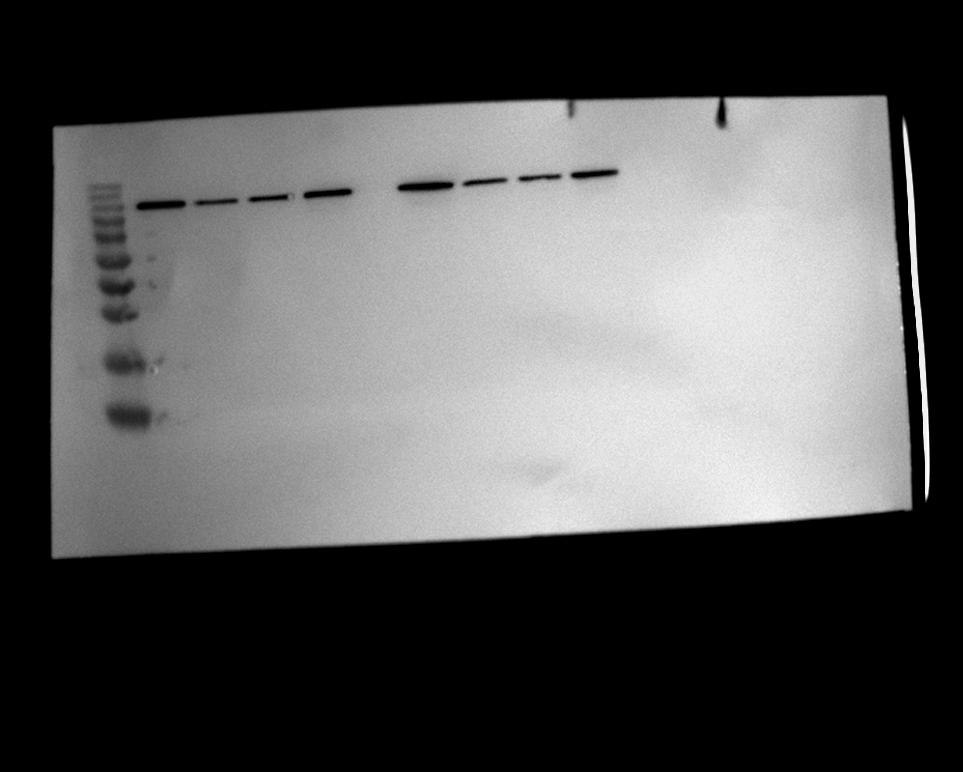

Supplement: Supplementary file 1 [file DataSheet1.zip › Western blot Original Images/Western blot Original Images 3/3/4H/1-HK2.tif]

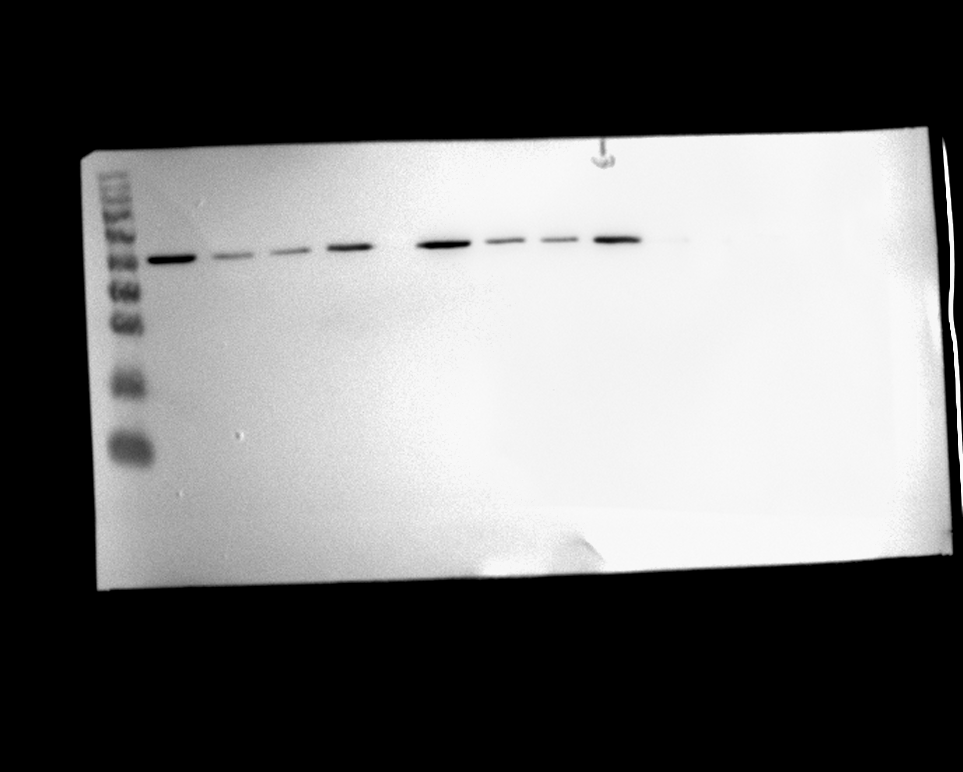

Supplement: Supplementary file 1 [file DataSheet1.zip › Western blot Original Images/Western blot Original Images 3/3/4H/2-PGK1.tif]

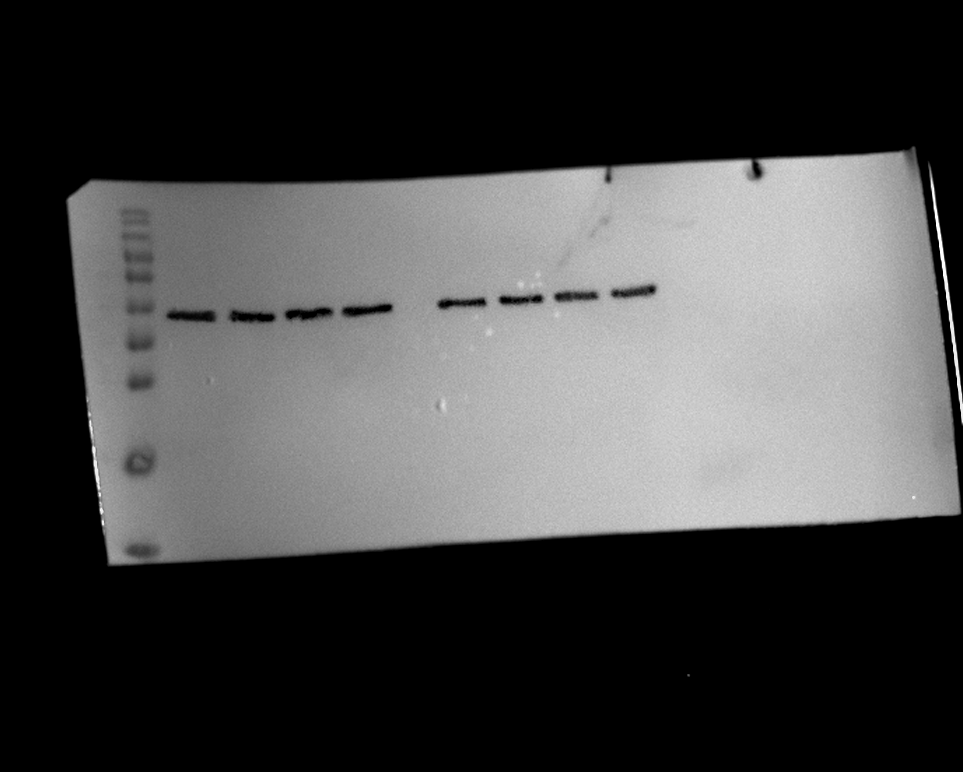

Supplement: Supplementary file 1 [file DataSheet1.zip › Western blot Original Images/Western blot Original Images 3/3/4H/3-β-actin.tif]

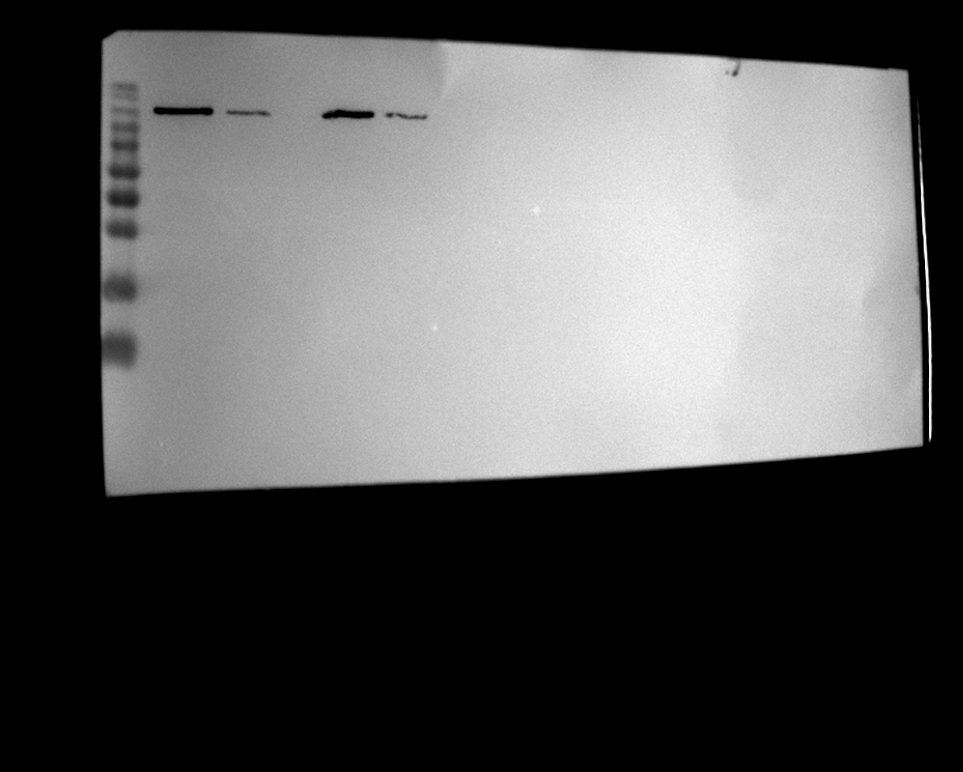

Supplement: Supplementary file 1 [file DataSheet1.zip › Western blot Original Images/Western blot Original Images 3/3/5B/1-NAT10.tif]

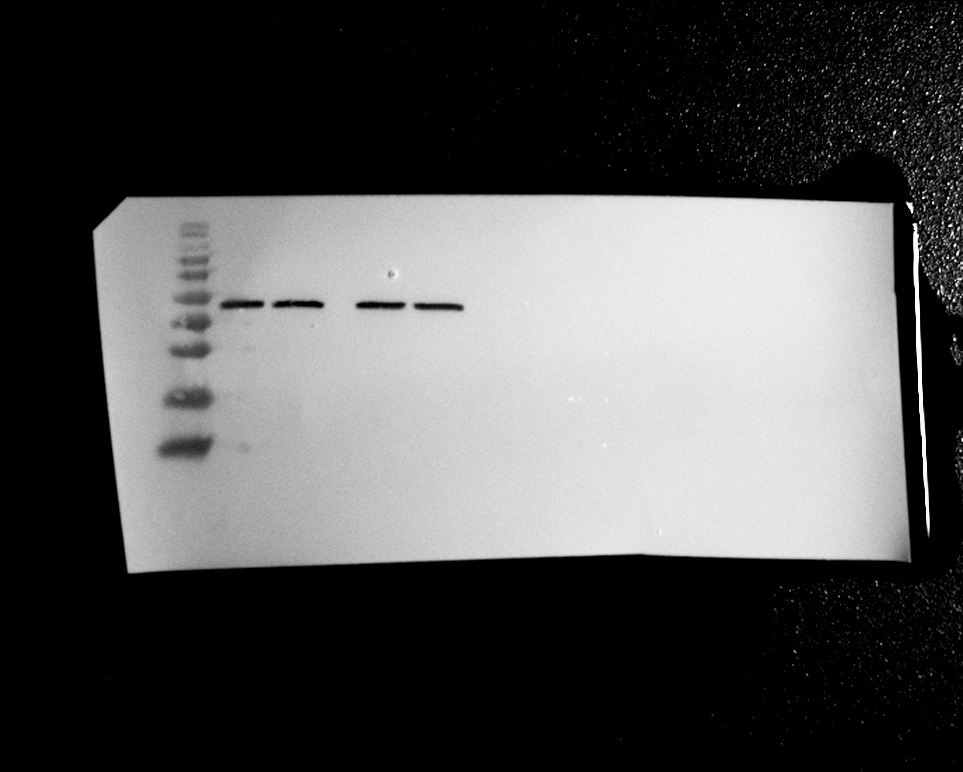

Supplement: Supplementary file 1 [file DataSheet1.zip › Western blot Original Images/Western blot Original Images 3/3/5B/2-β-actin.tif]

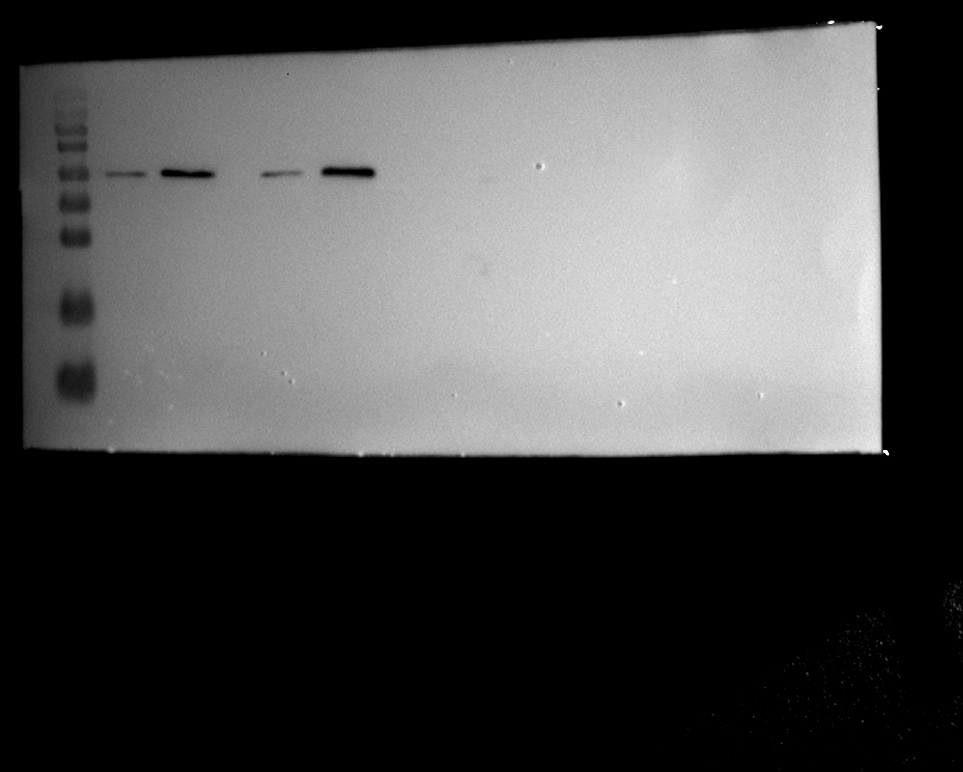

Supplement: Supplementary file 1 [file DataSheet1.zip › Western blot Original Images/Western blot Original Images 3/3/6B/1-PGK1.tif]

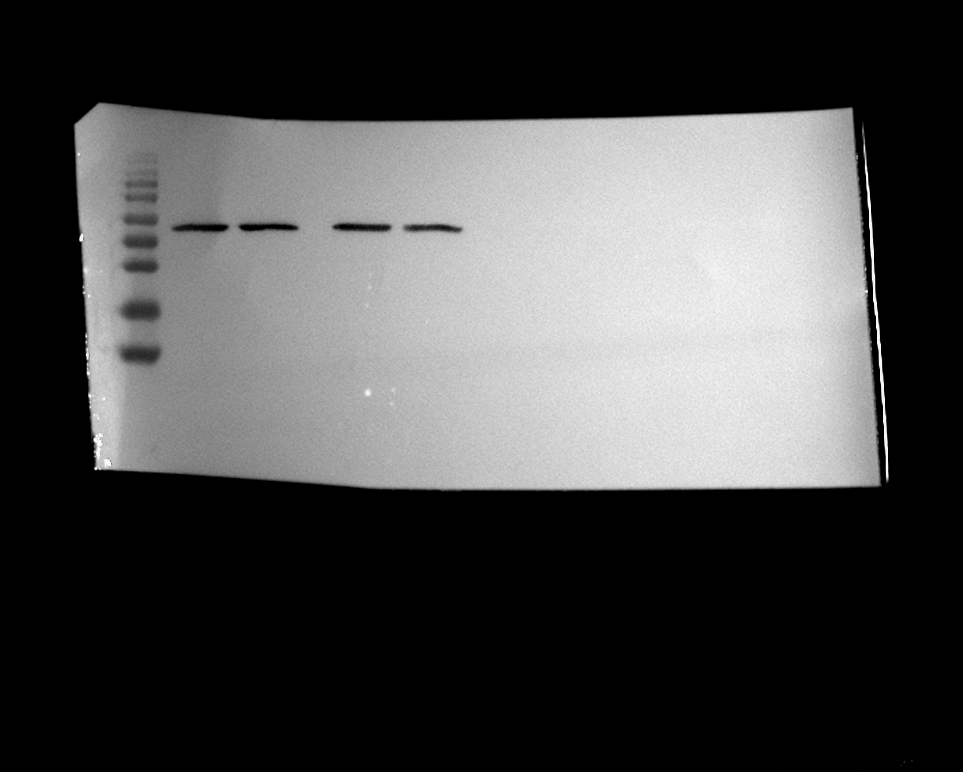

Supplement: Supplementary file 1 [file DataSheet1.zip › Western blot Original Images/Western blot Original Images 3/3/6B/2-β-actin.tif]

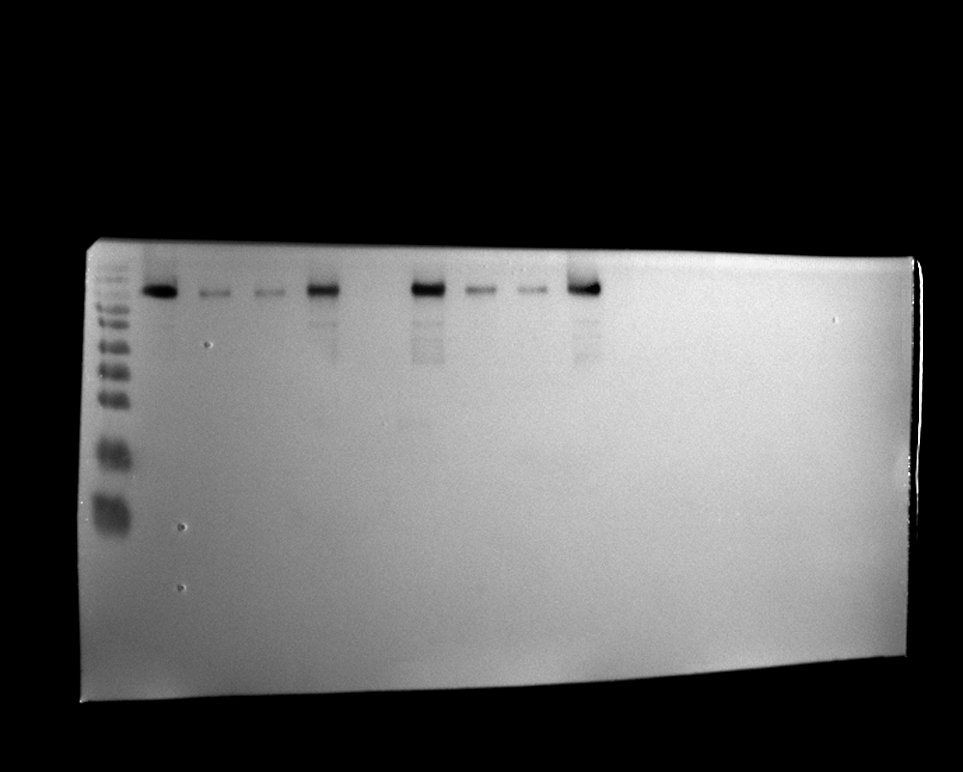

Supplement: Supplementary file 1 [file DataSheet1.zip › Western blot Original Images/Western blot Original Images 3/3/6H/1-HK2.tif]

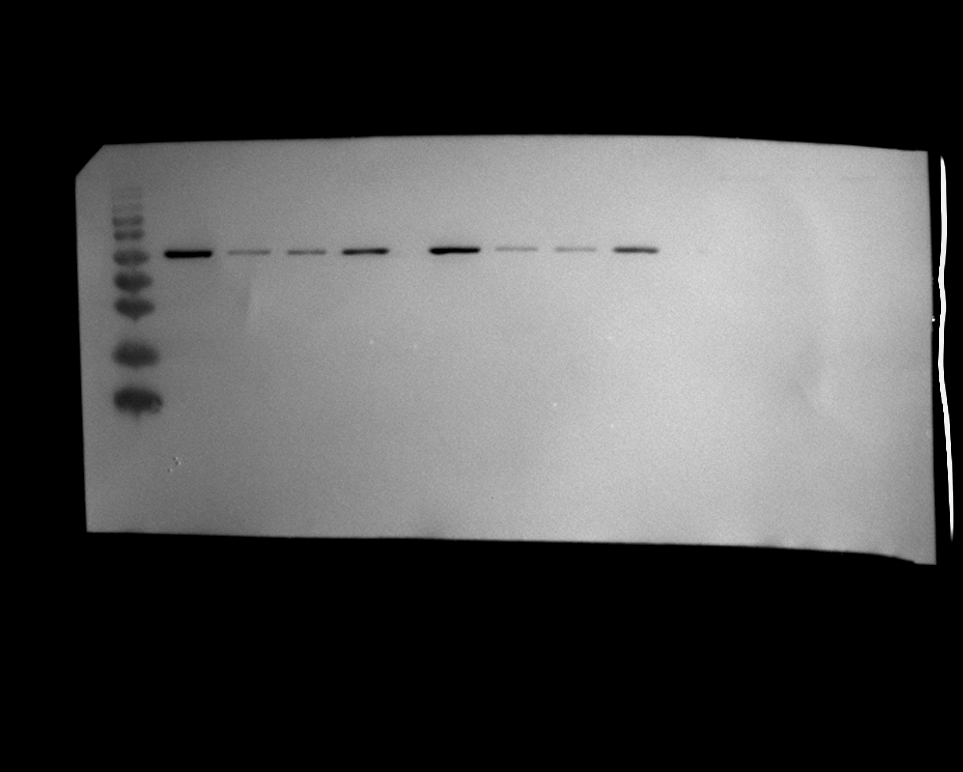

Supplement: Supplementary file 1 [file DataSheet1.zip › Western blot Original Images/Western blot Original Images 3/3/6H/2-PGK1.tif]

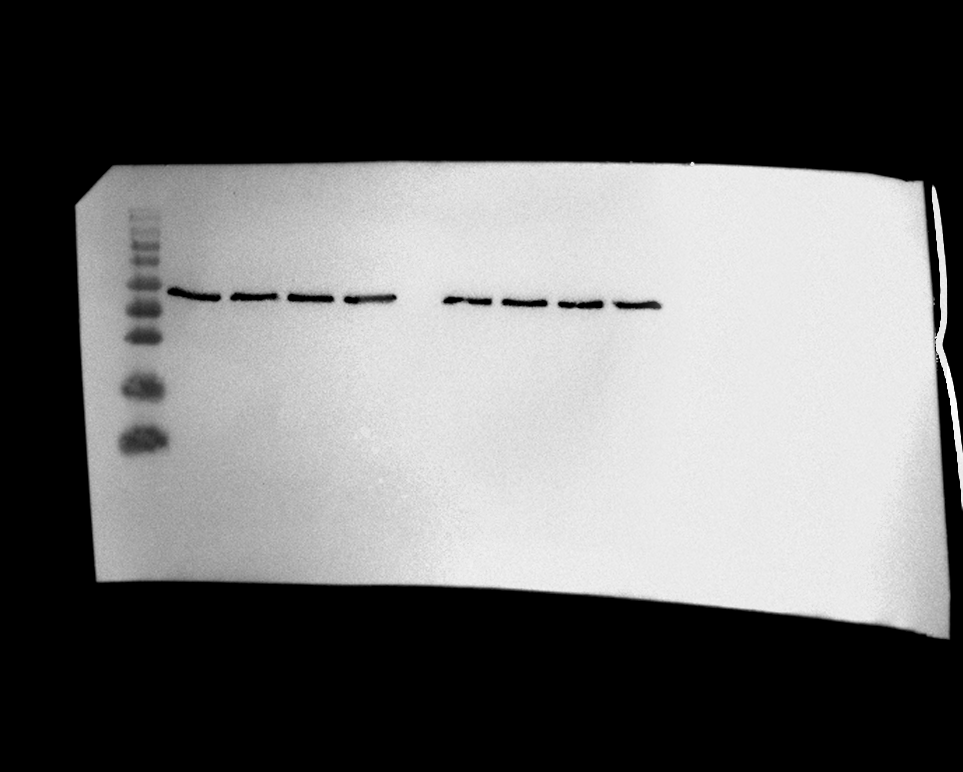

Supplement: Supplementary file 1 [file DataSheet1.zip › Western blot Original Images/Western blot Original Images 3/3/6H/3-β-actin.tif]

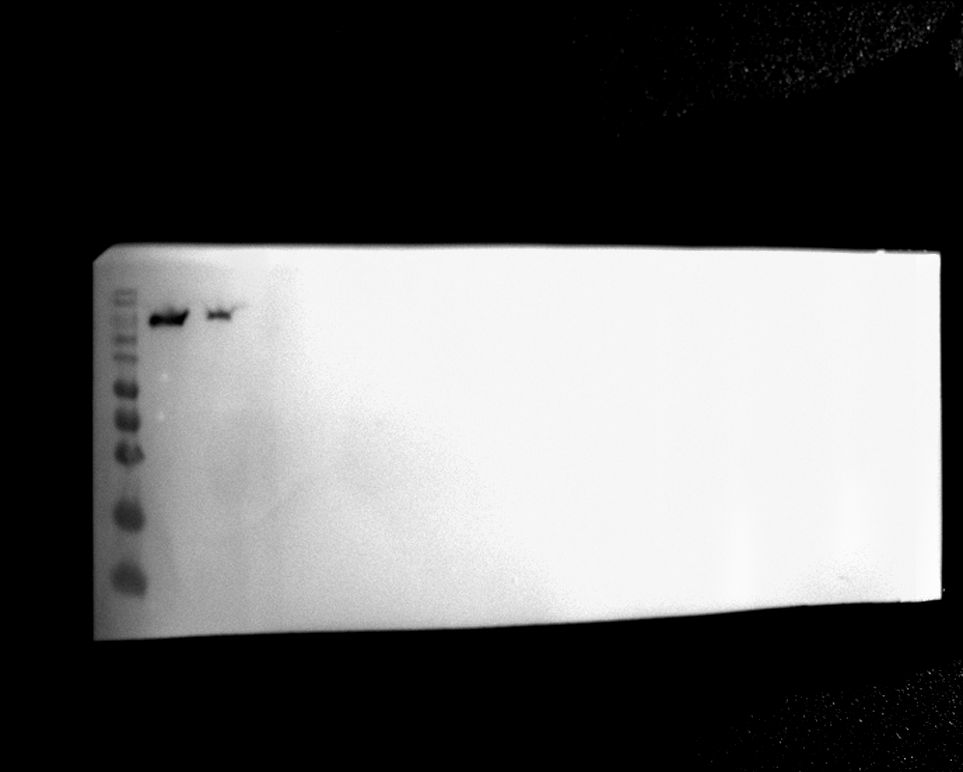

Supplement: Supplementary file 1 [file DataSheet1.zip › Western blot Original Images/Western blot Original Images 3/3/7E/1-HK2.tif]

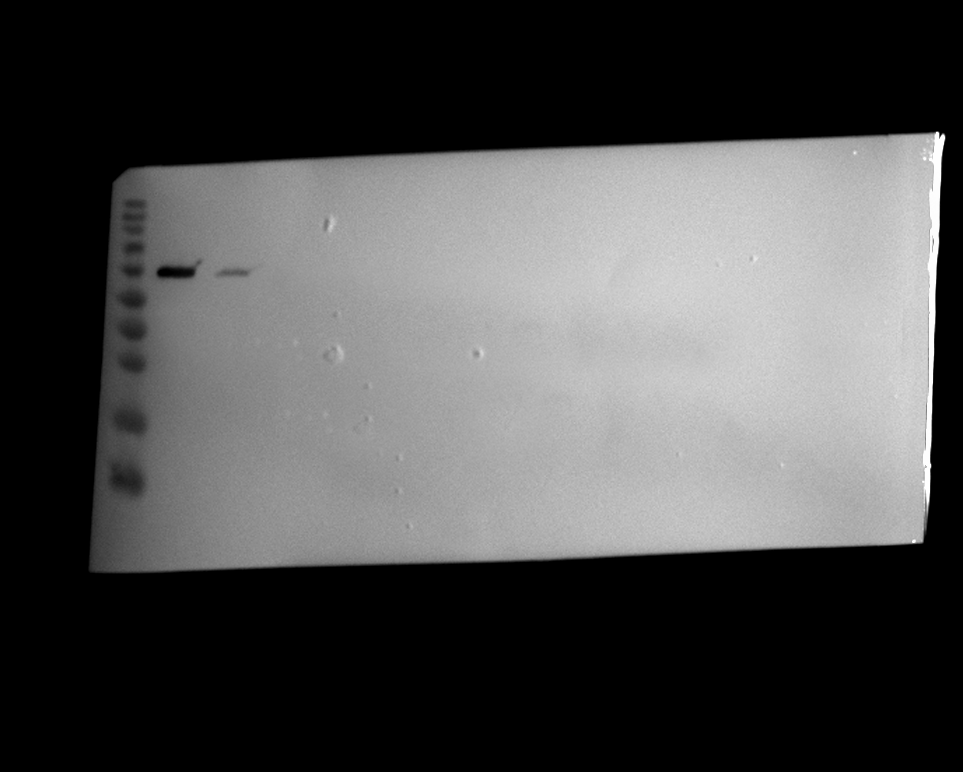

Supplement: Supplementary file 1 [file DataSheet1.zip › Western blot Original Images/Western blot Original Images 3/3/7E/2-PKM2.tif]

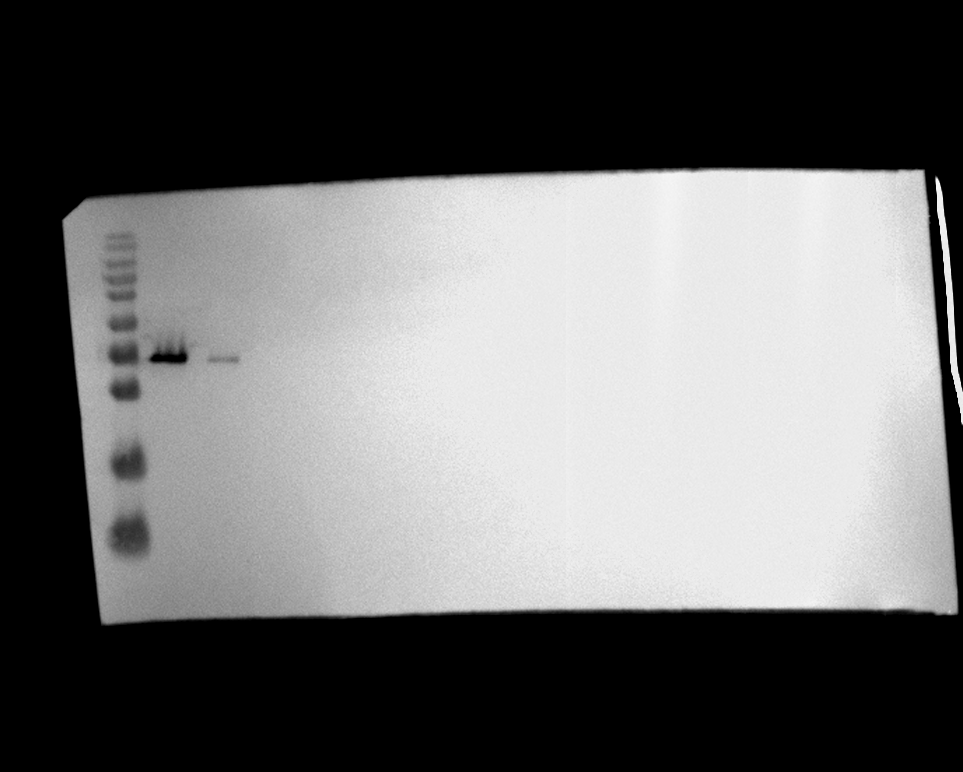

Supplement: Supplementary file 1 [file DataSheet1.zip › Western blot Original Images/Western blot Original Images 3/3/7E/3-LDHA.tif]

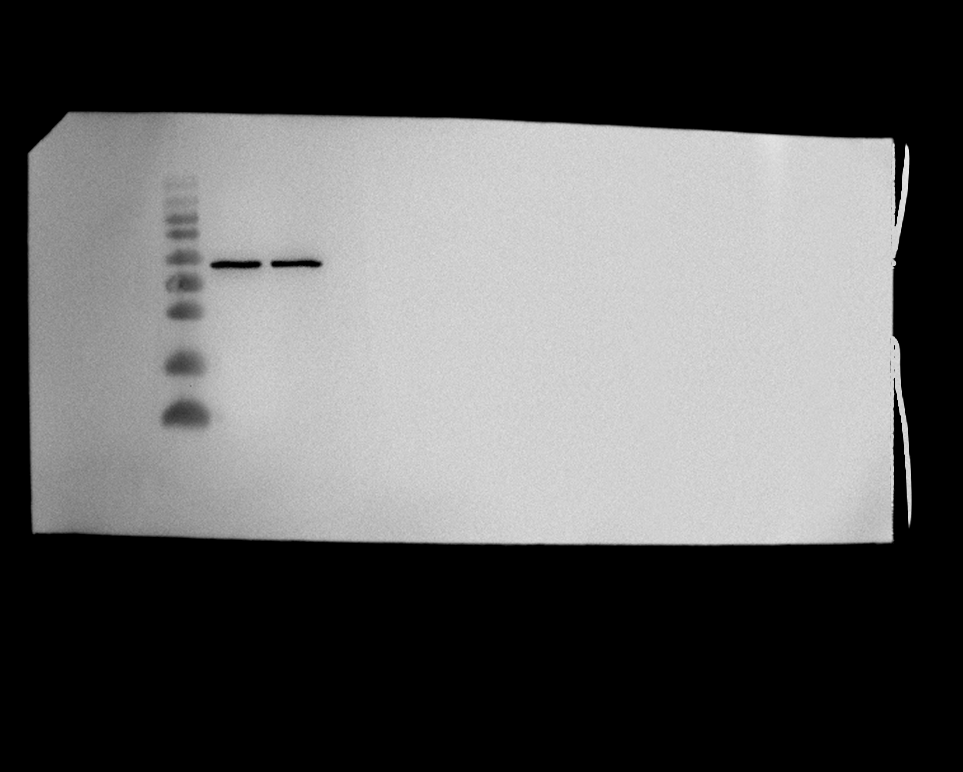

Supplement: Supplementary file 1 [file DataSheet1.zip › Western blot Original Images/Western blot Original Images 3/3/7E/4-β-actin.tif]

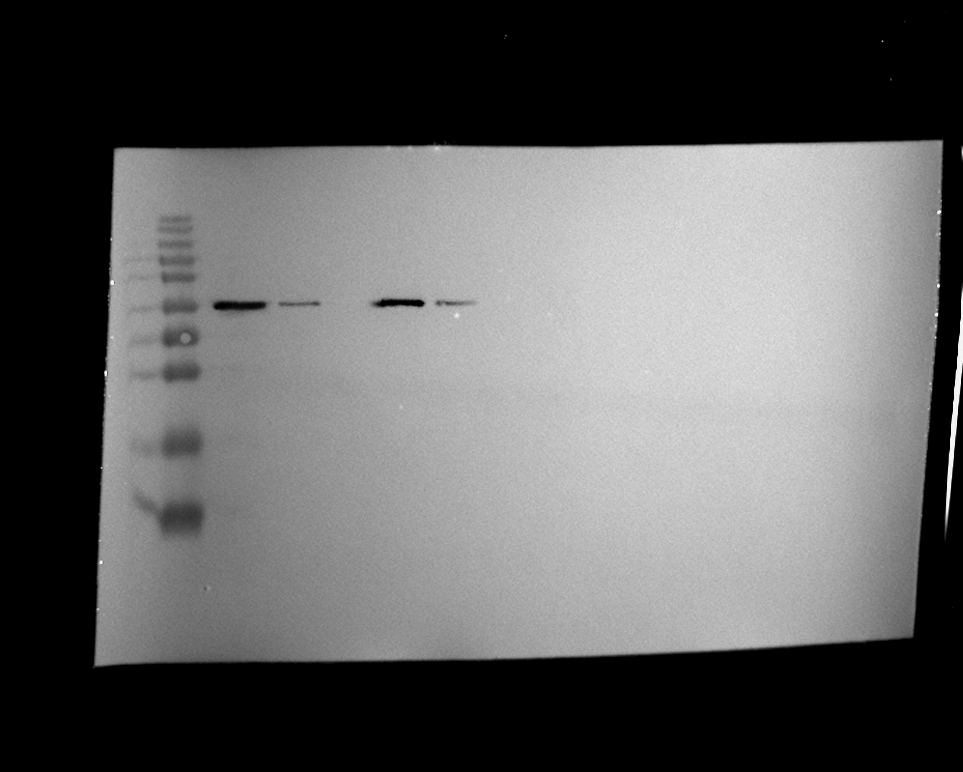

Supplement: Supplementary file 1 [file DataSheet1.zip › Western blot Original Images/Western blot Original Images 3/3/S1/1-PGK1.tif]

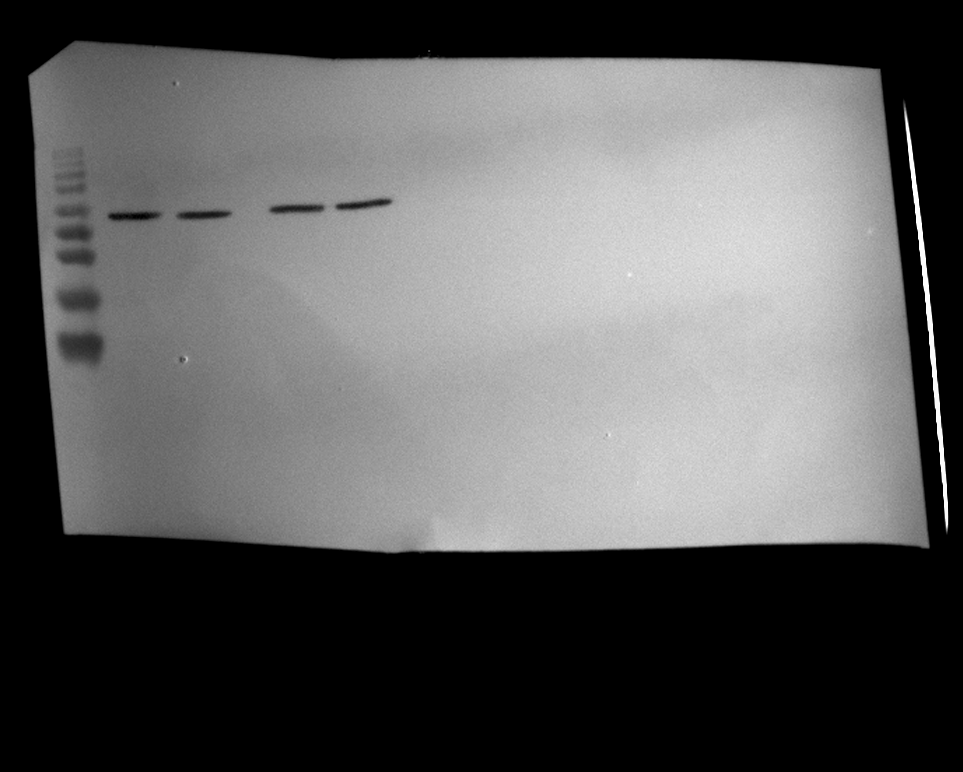

Supplement: Supplementary file 1 [file DataSheet1.zip › Western blot Original Images/Western blot Original Images 3/3/S1/2-β-actin.tif]

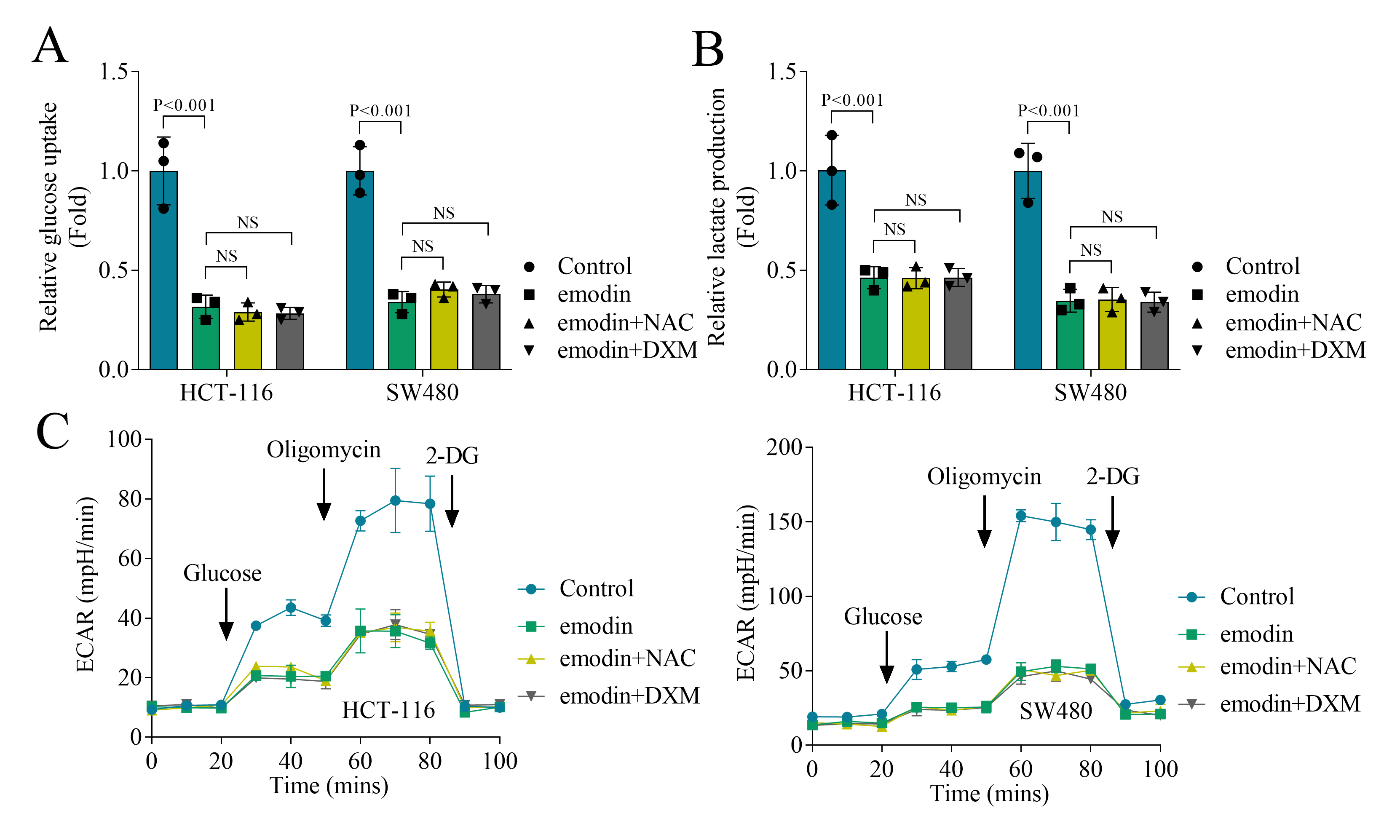

Supplement: Supplementary Figure 1 — Effects of NAC and DXM on glucose uptake, lactate production and ECAR in colon cancer cells. (A) Glucose uptake was evaluated using a glucose uptake assay kit. (B) Lactate production was evaluated using a lactate content detection kit. (C) Real-time cell metabolism was assessed by measuring ECAR. N = 3 per group. Data analysis was performed using ANOVA. [file Image1.jpeg]

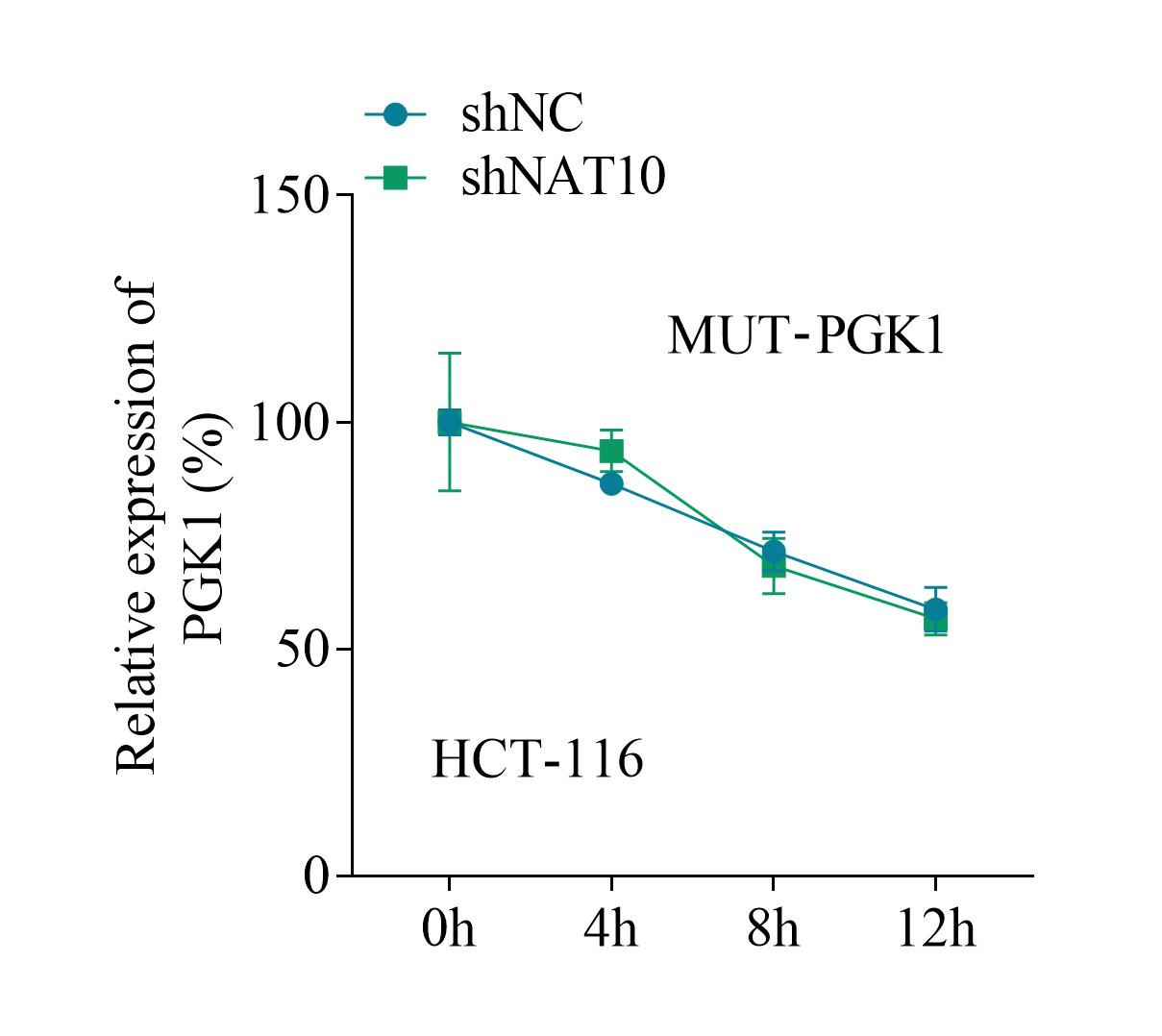

Supplement: Supplementary Figure 2 — The stability of PGK1 mRNA with ac4C site mutation. The stability of PGK1 mRNA was measured by qPCR after HCT-116 cells treated with 5 μg/mL actinomycin D for 0, 4, 8, and 12 h. N = 3 per group. Data analysis was performed using ANOVA. [file Image2.jpeg]

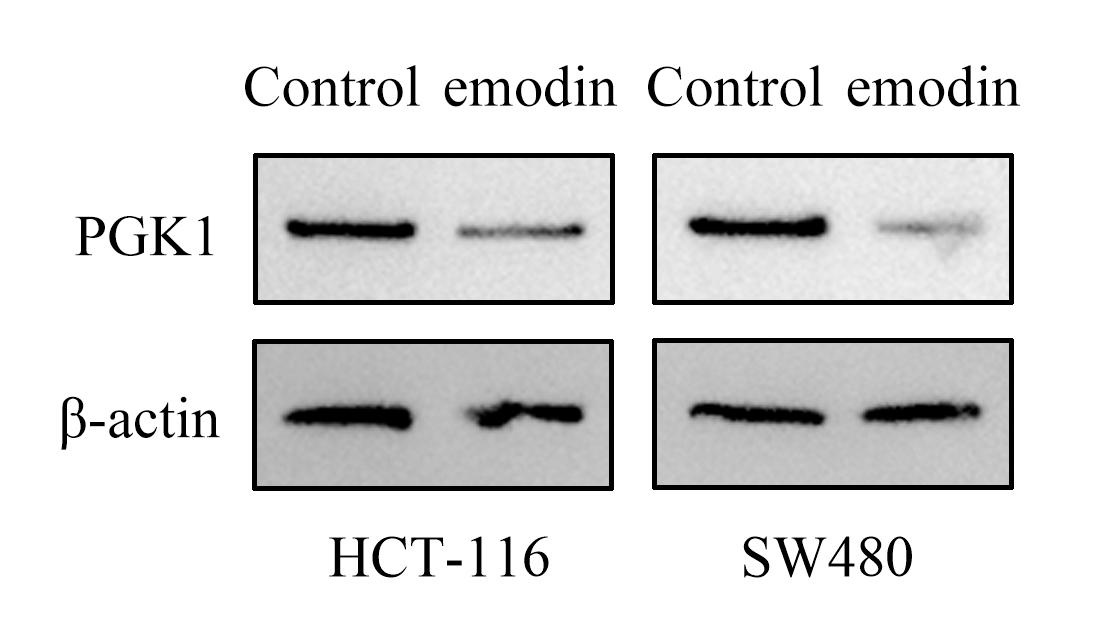

Supplement: Supplementary Figure 3 — The protein levels of PGK1 in HCT-116 and SW480 cells. The protein levels of PGK1 in HCT-116 and SW480 cells were detected by western blot. N = 3 per group. [file Image3.jpeg]
